# Redox-Switchable Aromaticity in a Helically Extended Indeno[2,1-*c*]fluorene

**DOI:** 10.1021/jacs.4c04191

**Published:** 2024-07-02

**Authors:** Eric Sidler, Robert Hein, Daniel Doellerer, Ben L. Feringa

**Affiliations:** Stratingh Institute for Chemistry, University of Groningen, Nijenborgh 4, 9747 AG Groningen, The Netherlands

## Abstract

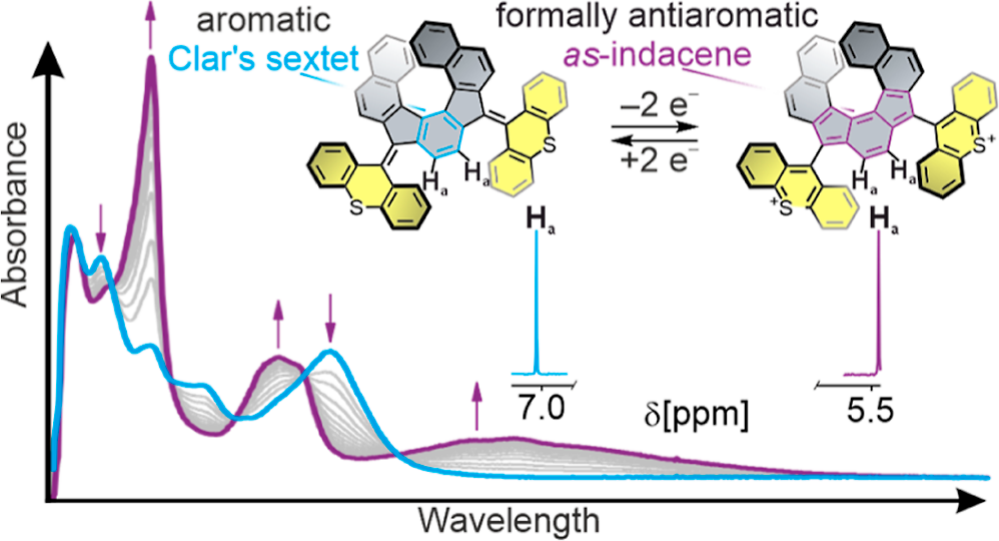

Molecular switches have received major attention to enable the reversible modulation of various molecular properties and have been extensively used as trigger elements in diverse fields, including molecular machines, responsive materials, and photopharmacology. Antiaromaticity is a fascinating property that has attracted not only significant fundamental interest but is also increasingly relevant in different applications, in particular organic (opto)electronics. However, designing systems in which (anti)aromaticity can be judiciously and reversibly switched ON and OFF remains challenging. Herein, we report a helicene featuring an indenofluorene-bridged bisthioxanthylidene as a novel switch wherein a simultaneous two-electron (electro)chemical redox process allows highly reversible modulation of its (anti)aromatic character. Specifically, the two thioxanthylidene rotors, attached to the initially aromatic indenofluorene scaffold via overcrowded alkenes, adopt an *anti*-folded structure, which upon oxidation convert to singly bonded, twisted conformations. This is not only associated with significant (chir)optical changes but importantly also results in formation of the fully conjugated, formally antiaromatic *as*-indacene motif in the helical core of the switch. This process proceeds without the buildup of radical cation intermediates and thus enables highly reversible switching of molecular geometry, aromaticity, absorbance, and chiral expression under ambient conditions, as evidenced by NMR, UV–vis, CD, and (spectro)electrochemical analyses, supported by DFT calculations. We expect this concept to be extendable to a wide range of robust antiaromatic–aromatic switches and to provide a basis for modulation of the structure and properties of these fascinating inherently chiral polycyclic π-scaffolds.

## Introduction

As one of the core concepts of chemistry, the study of aromaticity has remained at the forefront of contemporary research, with increasing focus on the investigation of systems beyond regular Hückel aromaticity.^1−6^ For example, there is growing interest in the development of (formally) antiaromatic polycyclic π-scaffolds, not only in the contex t of fundamental studies but also due to their unique (opto)electronic properties. Indenofluorenes (IFs) have recently received significant interest in this context, as this class of nonalternant polycyclic hydrocarbons bears, in its fully conjugated state, *s*- or *as*-indacene cores with 12 π electrons.^7^ This is associated with a range of striking properties, such as low reduction potentials, small energy bandgaps, and intense colors, which evidently harbor a large number of potential applications, relevant for, e.g., optoelectronic devices and organic solar cells.^8,9^ As a result, numerous (fully conjugated) IFs have been developed in the past decade,^10−12^ inspired by seminal work by Haley and co-workers on the synthesis of antiaromatic indeno[1,2-*b*]fluorenes.^13^ Various extended and regioisomeric IFs were reported;^14−16^ however, their synthesis can often be challenging. Conceivably, reversible in situ generation of the conjugated indacene core would provide convenient access to this antiaromatic motif from more accessible/stable precursors with the attractive feature of switching (anti)aromaticity.

Modulation of various molecular properties, including, in principle, (anti)aromaticity can be achieved using molecular switches driven by various stimuli.^17−21^ For example, it was shown that photoswitching^22−24^ of a biphenylene-diarylethene allows to induce significant and reversible changes in the antiaromatic character of the central biphenylene motif by modulation of the conjugation pattern.^25^ Similarly, switching between the open- and closed-shell states of an indacene core has recently been observed by changing the adsorption site of an on-surface synthesized unsubstituted indeno[1,2-*a*]fluorene.^26^

Alternatively, antiaromatic (IF) motifs can be generated by oxidation or reduction of more stable and readily accessible aromatic precursors, and is, in principle reversible.^27−34^ However, this almost always proceeds via intermediate radical states that are typically unstable, potentially leading to undesired side reactions.

**Figure 1:**
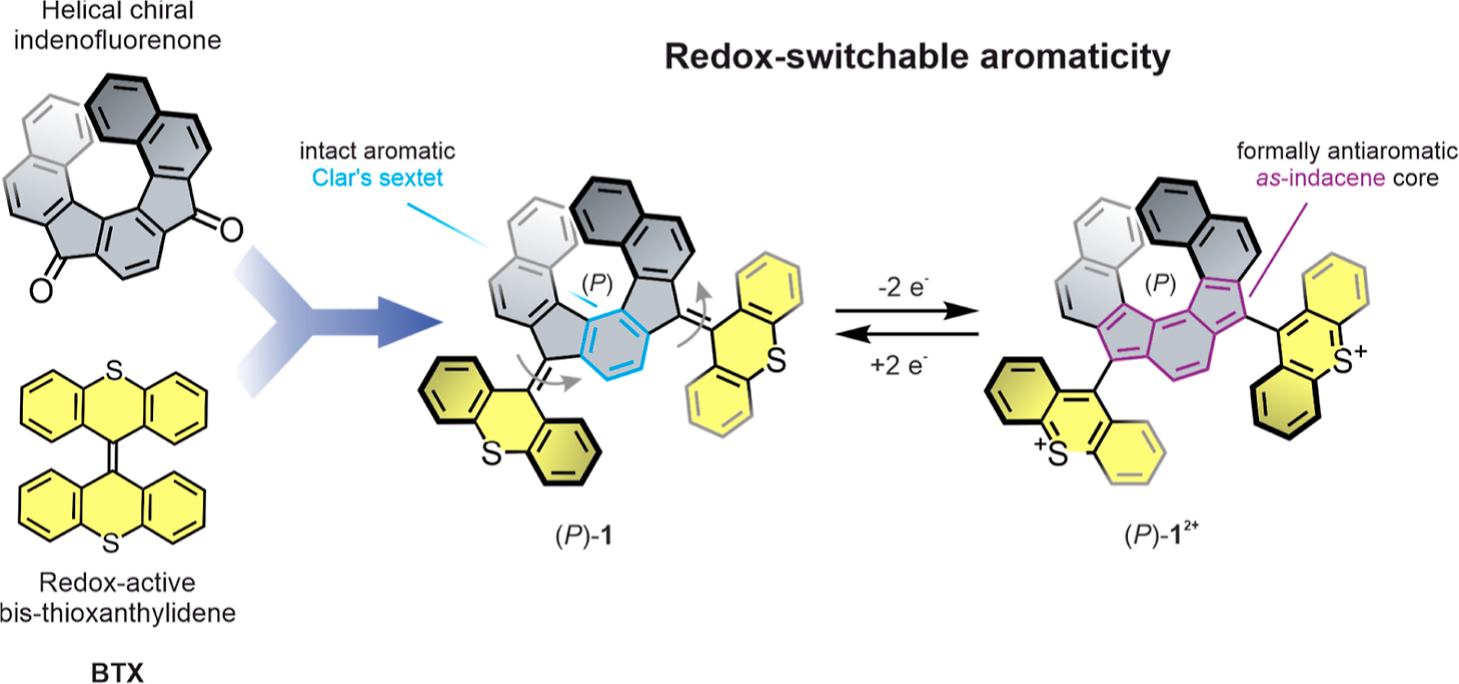
Helical extension of the BTX redox switch affords the novel, chiral switch **1** in which a formally antiaromatic as-indacene core can be conveniently, and highly reversibly, generated by simultaneous two-electron oxidation. This process is also associated with significant conformational rearrangements of the thioxanthylium rotors along with (chir)optical changes.

This problem is circumvented in dynamic redox (dyrex) switches, wherein two-electron redox processes are associated with significant geometric rearrangements.^35−37^ This has been exploited in dyrex switches, in which reversible C–C single or C=C double-bond formation/breaking via two-electron oxidations gives rise to not only significant conformational transformations but also changes in polarity, absorbance, and luminescence.^38−44^ For example, we have pioneered the use of the overcrowded alkene bisthioxanthylidene (**BTX**, Figure 1)^41,45−47^ and derivatives thereof^48^ as highly versatile redox and photoresponsive switches with multiple stable and isolable (redox) states.

We surmised that this dyrex concept can also be extended to the switching of antiaromaticity. To this end, we designed the novel, inherently chiral redox switch **1** that merges a helicene with an overcrowded alkene motif (Figure 1). More specifically, an extended, helical chiral dibenzo-indeno[2,1-*c*]fluorene core bridges two redox-active thioxanthylidenes, which are attached on both five-membered rings of the IF via overcrowded alkene bonds. Upon simultaneous one-electron oxidation of both thioxanthylidene rotors, radical–radical recombination in the indenofluorene core induces formation of the antiaromatic *as*-indacene, while the thioxanthylium motifs, now attached via C–C single bonds, rotate to alleviate crowding while also sterically shielding the *as*-indacene. This process, involving large geometrical changes, also coincides with intense (chir)optical changes as described and analyzed in detail using X-ray crystallography, nuclear magnetic resonance (NMR), UV–vis, circular dichroism (CD) spectroscopy, and spectroelectrochemistry. Importantly, these changes are highly reversible, presenting, to the best of our knowledge, the first example of a robust IF/indacene (anti)aromaticity switch.

## Results and Discussion

**Figure 2:**
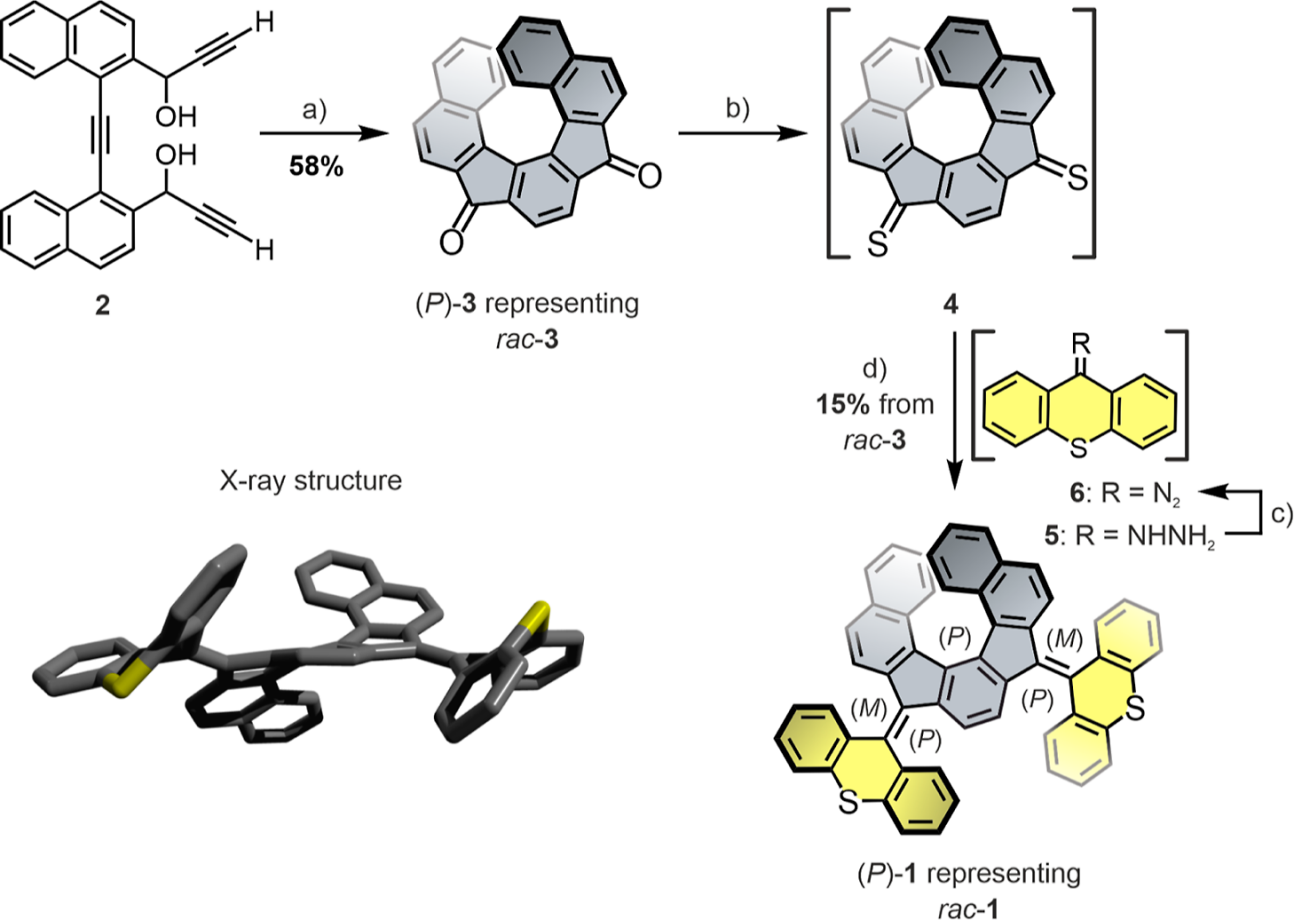
Synthetic scheme and crystal structure of *rac*-**1**. Conditions: (a) (1) Ag_2_CO_3_, [RhCl(PPh_3_)_3_], THF, mw, 180 °C, 1.5 h and (2) pyridinium chlorochromate, Celite, CH_2_Cl_2_, rt, 3 h. (b) Lawesson’s reagent, toluene, reflux, 1.5 h. (c) Ag_2_O, KOH (sat. in methanol), MgSO_4_, diethyl ether, 0 °C, 45 min. (d) HMPT, toluene, diethyl ether, rt, 15 min.

The synthesis of the desired switch *rac*-**1** is displayed in Figure 2. Starting from literature-reported diol **2**,^49^ a microwave-assisted rhodium-catalyzed cyclotrimerization^50^ yielded diketone **3** as a racemic mixture (*rac*-**3**) in 58% yield. The analytical data of *rac*-**3** matched literature reports, where **3** was obtained via an asymmetric synthesis route to yield enantioenriched samples.^49^ As we anticipated chiral separation to afford pure enantiomers instead of the reported scalemic mixture, we performed the reaction to obtain a racemate. Dithioketone **4**, which proved to be unstable in the solid state, was synthesized in situ and directly subjected to our standard Barton–Kellogg olefination procedure with diazo **6** that was in situ generated from hydrazone **5**.^51^ Desulfurization with hexamethylphosphorous triamide (HMPT) afforded the desired overcrowded alkene *rac*-**1** in 15% yield from *rac*-**3**.

**Figure 3:**
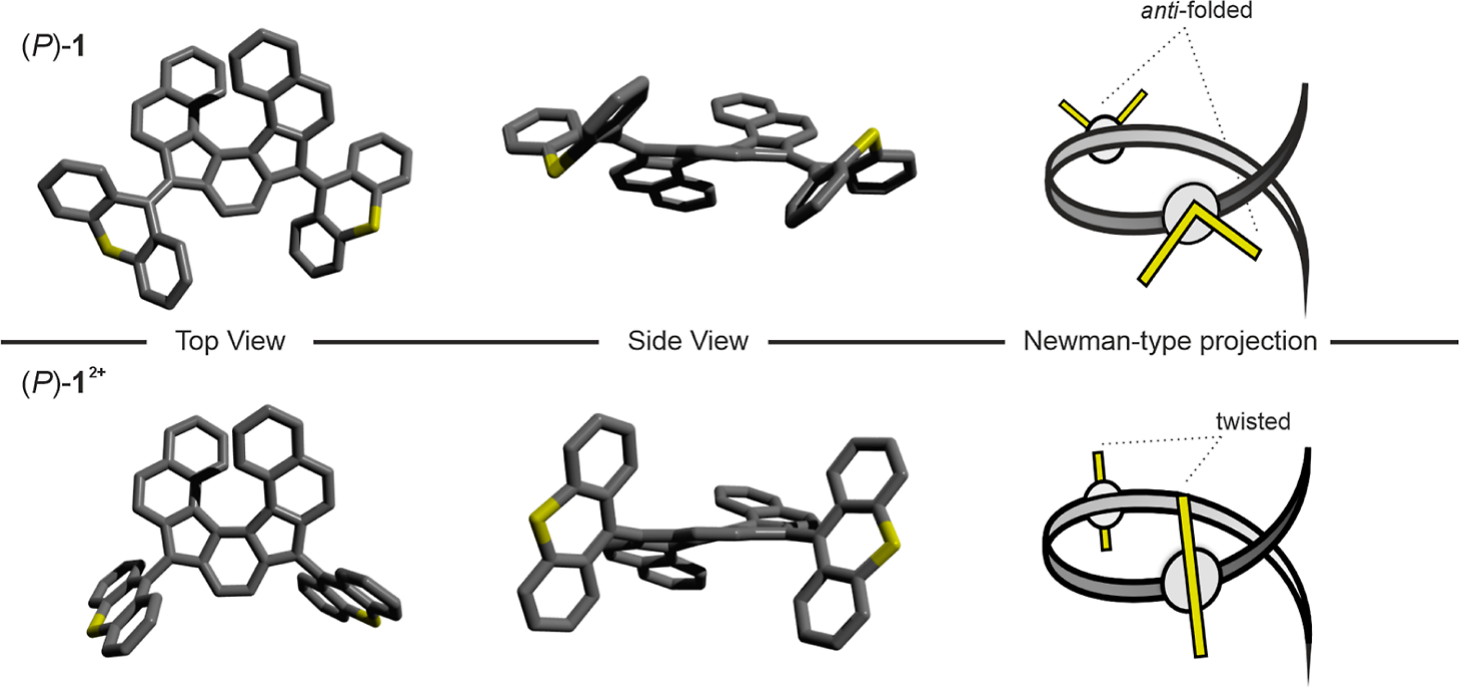
Top and side view of the DFT-optimized geometries of (*P*)-**1** (top) and (*P*)-**1**^**2+**^ (bottom), as well as illustration of the Newman-type projection, indicating an *anti*-folded and twisted structure for both rotors in (*P*)-**1** and (*P*)-**1**^**2+**^, respectively. The calculations were performed at the r^2^SCAN-3c/CPCM(CH_2_Cl_2_) level of theory.

The target compound *rac*-**1** was fully characterized by NMR spectroscopy and high-resolution mass spectrometry (HR-MS) (Figures S2 and S3). The ^1^H NMR spectrum displays only 15 proton signals in total. Furthermore, the presence of a singlet for the protons of the central phenylene in the helical indenofluorene indicates a *C*_2_-symmetry axis dissecting the helicene. Red single crystals suitable for X-ray crystallography were obtained by slow diffusion of a methanol top layer into *rac*-**1** in CD_2_Cl_2_. The crystal structure (Figure 2, bottom left) revealed a monoclinic unit cell containing each enantiomer ((*P*)-**1** and (*M*)-**1**) twice (see also Figures S4 and S5). In the solid state, both thioxanthylidene rotors are folded toward opposed sides of the central helix by adopting an *anti*-folded structure^41,48,52^ with respect to the pitch of the helical indenofluorene. This conformation is in good agreement with the *C*_2_ symmetry indicated by the NMR spectrum. Geometry optimizations using density functional theory (DFT) at a r^2^SCAN-3c/CPCM(CH_2_Cl_2_)^53,54^ level of theory were performed for (*P*)-**1** (Figure 3, top), matching well with the crystal structure. Computational conformational analysis revealed a range of other possible folded or twisted rotor conformers, all of which are however significantly higher in energy and inaccessible due to high transition state energies, see Figure S17.

Taken together, these results confirm the formation of four new helices in the fjord regions of the overcrowded alkene moieties during the Barton–Kellogg reaction. Notably, the configuration of all newly formed helices is hereby induced and predetermined by the stable configuration of the central helical indenofluorene (see stereochemical descriptors in Figure 2). In other words, we achieved stereoselective formation of four new helical substructures using a twofold coupling to a helicene core.

Upon twofold oxidation, the rotors are expected to be planarized and the overcrowded alkene bond to be transformed to a single bond (Figure 1), leading to the out-of-plane rotation of the thioxanthylium motifs to a doubly twisted conformation, which is in good agreement with the DFT-optimized structure of (*P*)-**1**^**2+**^ (Figure 3, bottom).

**Figure 4:**
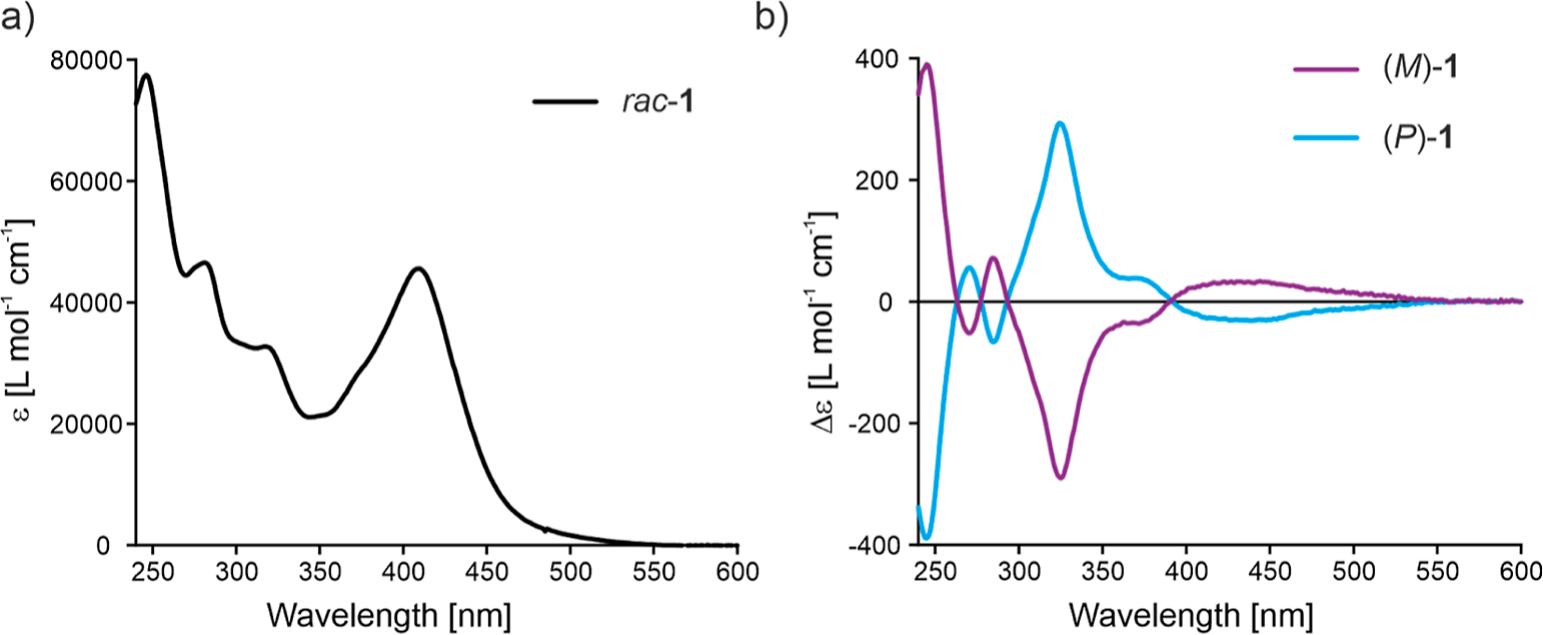
(a) UV–vis spectrum of *rac*-**1** in CH_2_Cl_2_ (*c* ∼ 10^–6^ M). (b) CD spectra of (*M*)-**1** (purple) and (*P*)-**1** (blue) in CH_2_Cl_2_ (*c* ∼ 10^–6^ M). Enantiomers were assigned by comparison of the TD-DFT-calculated CD spectrum of (*P*)-**1**.

The UV–vis spectrum of *rac*-**1** is displayed in Figure 4a. The absorption spectrum shows a maximum at λ = 246 nm (ε_max_ = 77′500 L mol^–1^ cm^–1^) and two less intense transitions at λ = 281 nm (ε = 46′500 L mol^–1^ cm^–1^) and λ = 318 nm (ε = 32′700 L mol^–1^ cm^–1^). Furthermore, it features a strong and broad absorption well into the visible range, reaching a λ_max_ = 409 nm with a large absorption coefficient of ε = 45′500 L mol^–1^ cm ^–1^, in accordance with the orange color of the compound.

*Rac*-**1** was then subjected to high-performance liquid chromatography (HPLC) using CHIRALPAK IE as a chiral stationary phase. Good separation was achieved, which enabled isolation of pure (*P*)-**1** (99% ee) and (*M*)-**1** (98% ee) on a milligram scale (see Figures S8–S10 and associated explanations). CD spectroscopy of both enantiomers in CH_2_Cl_2_ gave rise to mirror image spectra (Figure 4b). The spectrum of (*P*)-**1** features intense Cotton bands at 245 nm (Δε = −389 L mol^–1^ cm^–1^) and 325 nm (Δε = 293 L mol^–1^ cm^–1^). In analogy to the absorption spectra, the CD spectra feature notable intensities until ∼550 nm for both (*P*)-**1** and (*M*)-**1**. We also calculated the absorption dissymmetry factor *g*_abs_ (*g*_abs_ = Δε/ε),^55^ which revealed large values of *g*_abs_ ∼ 1 × 10^–2^ for the transition at 325 nm for (*P*)-**1** (Figure S11). Moreover, the shape of the g_abs_ plots is comparable to the CD spectra, indicating similar relative contributions of the magnetic and electric transition dipole moments for the observed transitions. Full time-dependent DFT (TD-DFT) calculations at the B3LYP/6-311g*/CPCM(CH_2_Cl_2_)^54,56−58^ level of theory for (*P*)-**1** allowed assignment of the absolute configurations by simulation of its CD spectrum (Figure S18).

**Figure 5:**
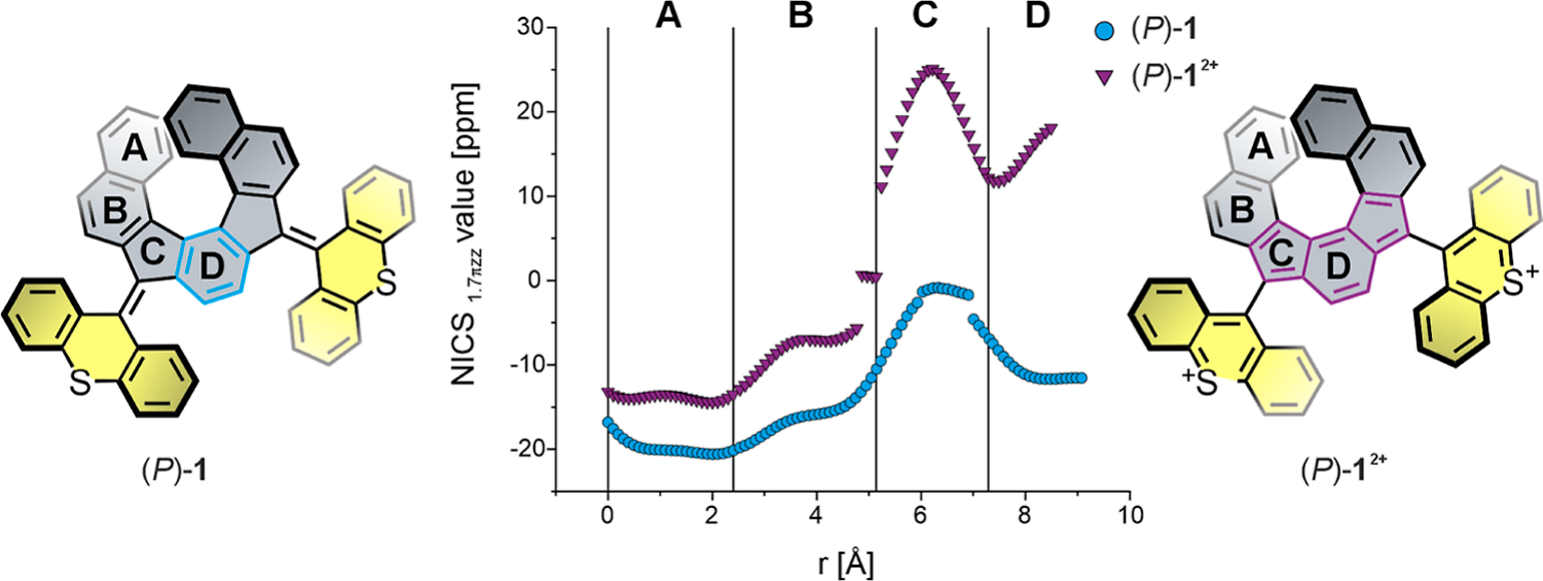
NICS_1.7πzz_-XY scan of (*P*)-**1** (blue) and (*P*)-**1**^**2+**^ (purple). The scan was performed from the edge of ring A to the center of ring D.

To assess the degree of (anti)aromaticity of the helical core, a nucleus-independent chemical shift scan in the XY plane (NICS_1.7πZZ_-XY scan) was conducted on (*P*)-**1** and (*P*)-**1**^**2+**^ at the B3LYP/6-311+G* level of theory (see the Supporting Information for details).^59−62^ Considering the symmetry of **1**, only half of the molecule was scanned in both (*P*)-**1** and (*P*)-**1**^**2+**^ (from the edge of ring A to the center of ring D, see Figure 5).

For (*P*)-**1**, the scan reveals diamagnetic ring currents for rings A, B, and D and a negligible ring current for ring C, indicating aromaticity and nonaromaticity, respectively. In contrast, for (*P*)-**1**^**2+**^, the scan reveals strong paratropic ring currents for rings C and D, suggesting antiaromaticity in the *as*-indacene core (vide infra). This trend is consistent with the previously reported computational NICS-XY scan of the unsubstituted heptacyclic benzo-fused indeno[2,1-*c*]fluorene core.^10^

**Figure 6:**
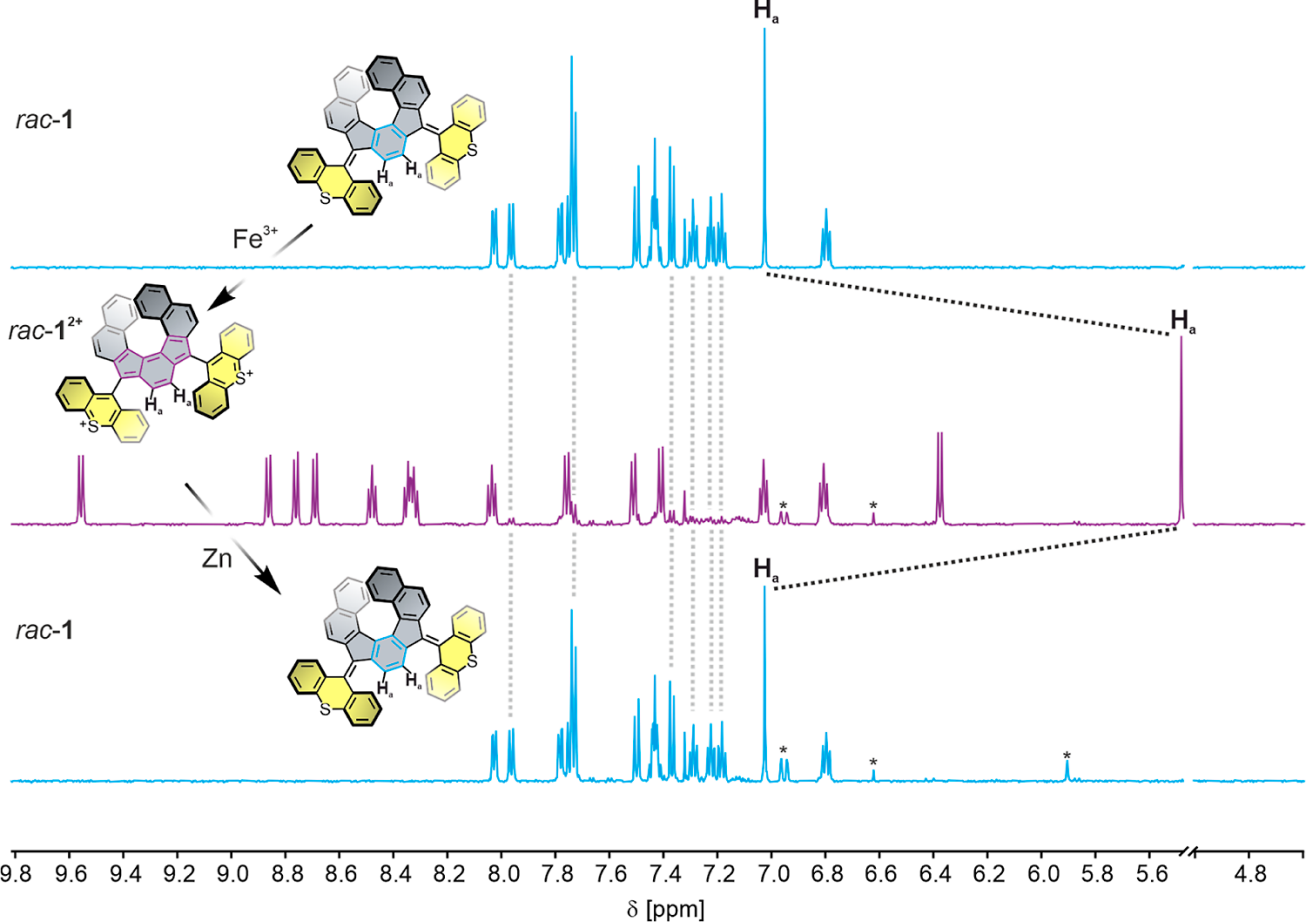
Stacked ^1^H NMR (600 MHz, 298 K) spectra of *rac*-**1** (top), *rac*-**1**^**2+**^ obtained by oxidation with Fe(ClO_4_)_3_ (middle), and rereduced *rac*-**1** obtained by reduction with Zn (bottom) in CD_2_Cl_2_. The black dotted lines illustrate the large shift of the central protons *H*_a_, and the gray dotted lines illustrate the remaining starting material. Side products are marked with an asterisk. The full spectra including proton assignments are shown in Figure S13.

To experimentally assess the redox switching capability of *rac*-**1**, initial chemical oxidation experiments of *rac*-**1** were performed on a small scale in CD_2_Cl_2_. Oxidation of *rac*-**1** with Fe(ClO_4_)_3_·*x*H_2_O under ambient conditions immediately resulted in a strong color change of the solution, going from yellow to deep purple. Concurrently, the ^1^H NMR spectrum revealed a relatively clean conversion to a closed-shell, diamagnetic species with the same number of distinct proton environments along with little residual starting material and very minor impurities (Figure 6, middle). This is consistent with retention of a *C*_2_-symmetry axis, which is in good agreement with two-electron oxidation to the dication *rac*-**1**^**2+**^, as well as the DFT-optimized twisted structure of the rotors in (*P*)-**1**^**2+**^ (Figure 3, bottom).

Notably, the singlet of proton H_a_ in the central phenylene, which resides at a chemical shift of δ = 7.03 ppm for *rac*-**1**, is drastically upfield shifted by 1.55 ppm to δ = 5.48 ppm upon oxidation to *rac*-**1**^**2+**^. This large shift suggests breaking up of the aromaticity of the central phenylene and formation of the formally antiaromatic *as*-indacene core, consistent with the conducted NICS-XY scan. In contrast, the downfield shift of various other peaks is in good agreement with formation of cationic, aromatized thioxanthylium rotors that are connected to the central helical scaffold via C–C single bonds.^41,45,48^ In addition, variable-temperature NMR (VT-NMR) studies of *rac*-**1**^**2+**^ in deuterated 1,1,2,2-tetrachloroethane (TCE-*d*_2_) confirm a low diradical character of the indeno[2,1-*c*]fluorene motif (Figure S15).

Pleasingly, upon chemical reduction of *rac*-**1**^**2+**^ with zinc, the initial NMR spectrum of the neutral *rac*-**1** was recovered, with negligible formation of other byproducts (Figure 6, bottom), indicative of a robust redox switching process. Similarly, *rac*-**1**^**2+**^ was generated by dissolving *rac*-**1** in deuterated trifluoroacetic acid (TFA-d) which induced slow oxidation to the dication, yielding a deep-purple solution. In this solution, *rac*-**1**^**2+**^ was stable over the course of multiple months at room temperature (see Figure S14 and associated discussion for more details). However, isolation of the pure dication salt by evaporation of the solvent was unsuccessful.

### Electrochemistry

**Figure 7:**
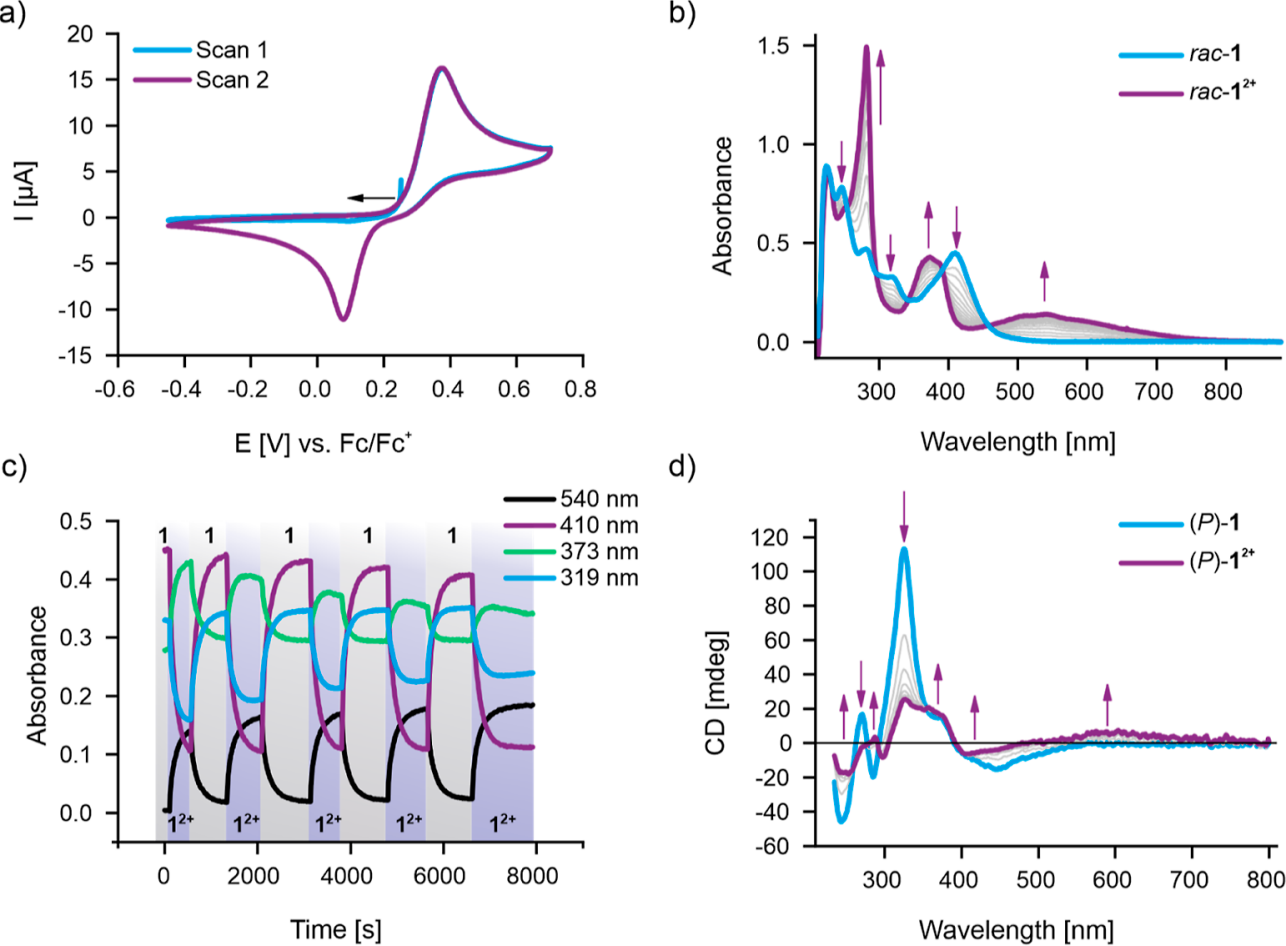
(a) CV of 0.5 mM *rac*-**1** in CH_2_Cl_2_, 100 mM TBAPF_6_ at ν = 100 mV/s. The black arrow indicates the starting point and initial direction of the first scan. (b) UV–vis spectra and (c) time traces of spectroelectrochemical interconversion of *rac*-**1**/*rac*-**1**^**2+**^ in CH_2_Cl_2_, 200 mM TBAPF_6_. The areas shaded in gray represent the reductive cycle (*E* = −0.45 V), while the blue areas represent oxidation (*E* = +0.70 V). (d) CD spectra of spectroelectrochemical conversion of (*P*)-**1** to (*P*)-**1**^**2+**^ in CH_2_Cl_2_, 200 mM TBAPF_6_. The corresponding time traces are shown in Figure S27.

The redox properties of *rac*-**1** were then studied in detail by cyclic voltammetry (CV) in CH_2_Cl_2_. As shown in Figure 7a, only one redox wave is observed in both anodic and cathodic scan directions with peak potentials of *E*_pa_ = +0.37 and *E*_pc_ = +0.08 V vs Fc/Fc^+^, respectively (at a scan rate of 100 mV/s). This corresponds to a moderate but notable hysteresis of 290 mV, which is in good agreement with the dynamic nature of the switching process, whereby simultaneous two-electron redox is associated with significant geometric rearrangements from a folded to a twisted state.^41,45^ This behavior is also observed in a range of related redox-active overcrowded alkenes,^43,48,63,64^ including the parent **BTX**, which, due to its more significant crowding in the fjord region, displays an even larger hysteresis (∼800 mV).^41,45^

The hysteretic nature of this process is also apparent in the first CV scan shown in Figure 7a. Specifically, no redox activity is observed in the potential window cathodic of the oxidation peak if no *rac*-**1**^**2+**^ was prior generated, confirming that *rac*-**1** and *rac*-**1**^**2+**^ do not represent a classic, electrochemically reversible redox couple.‡ Furthermore, this hysteretic two-electron redox behavior persists over a large range of different scan rates (25–1000 mV/s), as shown in Figure S21. The excellent linearity of the peak currents on the square root of the scan rate additionally confirms that all redox processes are diffusion controlled (Figure S22).

In analogy to related overcrowded alkene redox switches, it is possible that the oxidation of *rac*-**1** proceeds via an ECE mechanisms (electron transfer → chemical process (rearrangement) → electron transfer).^42,63^ Specifically, initial one-electron oxidation of one of the thioxanthylidene rotors transiently generates a radical cation *rac*-**1**^**+·**^, which quickly conformationally rearranges to a twisted structure that possesses a lower oxidation potential. The removal of an electron from this radical cation is at least as facile, or even more facile, than the initial oxidation and thus occurs immediately, thereby generating *rac*-**1**^**2+**^ (potential compression/inversion, Figure S23). However, alternative mechanisms in which a conformational rearrangement precedes electron transfer^43,65^ cannot be ruled out, as discussed in detail in the Supporting Information (Figures S24–S26). Regardless of the exact mechanism, oxidation (as well as reduction) proceeds without the buildup of the potentially reactive radical cation intermediate, an important feature of this system that undoubtedly contributes to its high degree of reversibility and stability (vide infra).

### Spectroelectrochemistry

To further characterize the redox switching properties of *rac*-**1**, UV–vis spectroelectrochemical studies were carried out. As shown in Figure 7b, bulk potential-controlled oxidation of the switch induced well-defined changes in the absorbance spectra that proceed via clear isosbestic points, which is in good agreement with direct conversion of *rac*-**1** to *rac*-**1**^**2+**^ without buildup of the radical cation intermediate *rac*-**1**^**+·**^. Specifically, upon oxidation, the initial band of the neutral switch at 410 nm completely disappeared, while strong, new absorbance peaks centered at 282 and 373 nm emerged, which can be attributed to the thioxanthylium motifs.^41,48^ Additionally, a somewhat weaker but very broad new band in the visible range appeared with a λ_max_ ∼ 540 nm, endowing *rac*-**1**^**2+**^ with a deep-purple coloration. The thioxanthylium cation also displays an absorbance band in this region, as observed in the red-colored **BTX**^**2+**^; however, its absorbance does not extend beyond 575 nm.^41,48^ In contrast, *rac*-**1**^**2+**^ shows significant absorbance until ∼750 nm, indicating that the antiaromatic helicene core significantly contributes to this new broad band. Indeed, *as*-indacenes display similar absorbance features in the visible region, further confirming the formation of this antiaromatic scaffold in *rac*-**1**^**2+**^.^10,11^ This was also corroborated by full TD-DFT calculations at the B3LYP/6-311g*/CPCM(CH_2_Cl_2_)^54,56−58^ level of theory. The red-shifted region of the calculated absorption spectrum of (*P*)-**1**^**2+**^ (Figure S19) is dominated by transitions involving occupied molecular orbitals (MOs) on the central helicene and unoccupied MOs on the cationic thioxanthylium rotors as well as the *as*-indacene core (Table S2 and Figure S20).

The absorption spectrum of the electrochemically generated dication is identical to that obtained by chemical oxidation, corroborating the formation of the same species (Figure S28). Upon reduction of the dication, the initial absorbance features of *rac*-**1** were quantitatively restored. In fact, over multiple successive redox cycles, only minimal fatigue was observed in the UV–vis spectra (Figure 7c), showcasing the robustness and high degree of reversibility of the redox switching.

To study the influence of oxidation on the chiral expression of the redox switch, we also conducted CD–spectroelectrochemical studies on (*P*)-**1**. As shown in Figure 7d, significant changes were observed for all Cotton bands upon oxidation. For example, the strongest, positive Cotton band of the neutral (*P*)-**1** at 325 nm largely decreases in intensity, while the negative band at 445 nm not only diminishes but also shifts to 408 nm. Additionally, in (*P*)-**1**^**2+**^, a new, broad, positive Cotton signal emerges at ∼605 nm. Importantly, these CD changes were remarkably reversible over multiple redox cycles, highlighting that both neutral and dicationic states of (*P*)-**1** do not racemize (Figure S27) at room temperature. This was further confirmed by CD measurement of (*M*)-**1** at 85 °C in toluene, whereby no racemization was observed (see Figure S12 and associated discussion).

## Conclusions

Herein, we showed that the helically extended bisthioxanthylidene **1** is a versatile, redox-responsive chiroptical switch that can reversibly interconvert aromatic and antiaromatic states within its heptacyclic indenofluorene core. The latter is easily accessible via two-electron chemical or electrochemical oxidation, generating the dicationic switch state **1**^**2+**^ that contains the formally antiaromatic *as*-indacene motif and is stable in solution over the course of months. This represents a rare example of the integration of this antiaromatic building block into an intrinsically chiral scaffold^30,31,66^ and enables switching of not only the (anti)aromatic character but also the chiroptical properties with high fidelity as demonstrated for enantiopure (*P*)-**1**.

The dynamic nature of the switching process, wherein the thioxanthylidene rotors undergo significant conformational changes from *anti*-folded to twisted upon oxidation, is hereby associated with a unique redox behavior in which both oxidation and reduction occur via virtually simultaneous two-electron transfer. Consequently, the buildup of the potentially reactive radical cation intermediate is circumvented, which undoubtedly contributes to the high stability and reversibility of the switching process, even under ambient conditions. As a result, **1** is a robust, redox-triggered chiroptical switch that undergoes well-defined and substantial changes in molecular geometry, aromaticity, absorbance, and chiral properties under ambient conditions. These responsive functions will undoubtedly be of significant interest across numerous applications, ranging from molecular switches and machines to optoelectronic devices. Moreover, we believe that this dynamic redox switching concept can be extended to a large range of related (nonalternant) structures, including other indenofluorene regioisomers, in which (anti)aromaticity can be judiciously switched in situ.

## Supporting Information Available

Further details on experimental procedures, NMR spectra, enantiomeric separation, DFT calculations, (spectro)electrochemical data, and mechanistic discussion (PDF)
XYZ files (ZIP)

## Footnotes

‡ It is important to note that although all individual redox processes for this switch are chemically irreversible (as they are associated with (directional) conformational changes), the overall switching between **1** and **1**^**2+**^ is fully reversible, see Figure 7c.
