# Supplementary material for: Redox-Switchable Aromaticity in a Helically Extended Indeno[2,1-c]fluorene
Source: J Am Chem Soc. 2024 Jul 2;146(28):19168–76. doi: 10.1021/jacs.4c04191 (PMC11258684; doi:10.1021/jacs.4c04191)
Supplement: Supplementary file 1 — ja4c04191_si_001.pdf [file ja4c04191_si_001.pdf]

Supporting Information

Redox-Switchable Aromaticity in a Helically Extended Indeno[2,1-*c*]fluorene

Eric Sidler,[a]† Robert Hein,[a]† Daniel Doellerer[a] and Ben L. Feringa*[a]

† Equally contributing first authors

*To whom correspondence should be addressed

b.l.feringa@rug.nl

[a] Stratingh Institute for Chemistry, University of Groningen, Nijenborgh 4, 9747 AG Groningen (The Netherlands)

Table of Contents

General Remarks 3

Synthesis & Characterization 4

X-Ray Crystallography (CCDC 2341064) 9

UV-Vis Dilution Series 12

HPLC Separation & g-Factor Plot..13

^{1}H NMR Chemical Redox Studies...16

DFT calculations 19

Electrochemical Measurements and DYREX Mechanism 25

Spectroelectrochemistry...31

General Remarks

All reagents were obtained from commercial sources and used as received without further purification. Dry solvents were obtained from a MBraun solvent purification system. Progress of the reactions was determined by TLC: silica gel 60, Merck, 0.25 mm. The TLC plates were visualized with ultraviolet (UV) light (λ = 254 nm or 355 nm). Microwave reactions were carried out using a CEM Discover SP synthesis system. Column chromatography was performed on a Biotage Selekt System. High Resolution Mass Spectrometry (HRMS) measurements were performed using an LTQ Orbitrap XL. NMR spectra were recorded on a Bruker Avance Neo with Cryoprobe Prodigy BBO (^{1}H: 600 MHz, ^{13}C: 151 MHz), a Varian Mercury Plus (^{1}H: 400 MHz) or an Agilent MR (^{1}H: 400 MHz) instrument. Chemical shifts (δ) are in parts per million (ppm) relative to TMS. For ^{1}H NMR spectroscopy, the splitting pattern of peaks is designated as follows: s (singlet), d (doublet), t (triplet), q (quartet), m (multiplet), dd (doublet of doublets), td (triplet of doublets), dq (quartet of doublets), and qt (quartet of triplets). Single-crystal X-ray diffraction measurements were performed on a Bruker-AXS D8 Venture diffractometer. UV/Vis absorption spectra were recorded on a Agilent Cary 8454 spectrophotometer in a 1 cm quartz cuvette. CD spectra were obtained on a Jasco J-815 spectropolarimeter. Electrochemical measurements were carried out with a Palmsense 4 potentiostat in a three-electrode setup comprising a Pt wire counter electrode, a non-aqueous Ag/$AgNO_3$ reference electrode (10 mM $AgNO_3$ in CH_3CN, 100 mM $TBAPF_6$) and a glassy carbon disk working electrode (3 mm diameter). Electrochemical grade $TBAPF_6$ was obtained from Sigma Aldrich. Spectroelectrochemistry was performed using a quartz spectroelectrochemical cell with 1 mm pathlength (ALS Japan). Solvents used for spectroscopic studies were of spectroscopic grade (UVASOL, Merck). Geometry optimizations and TD-DFT calculations were performed using the Orca 5.0.1. package.[1] The NICS-XY scan was calculated using the Gaussian 16 Rev. B.01 software package.[2]

Synthesis & Characterization

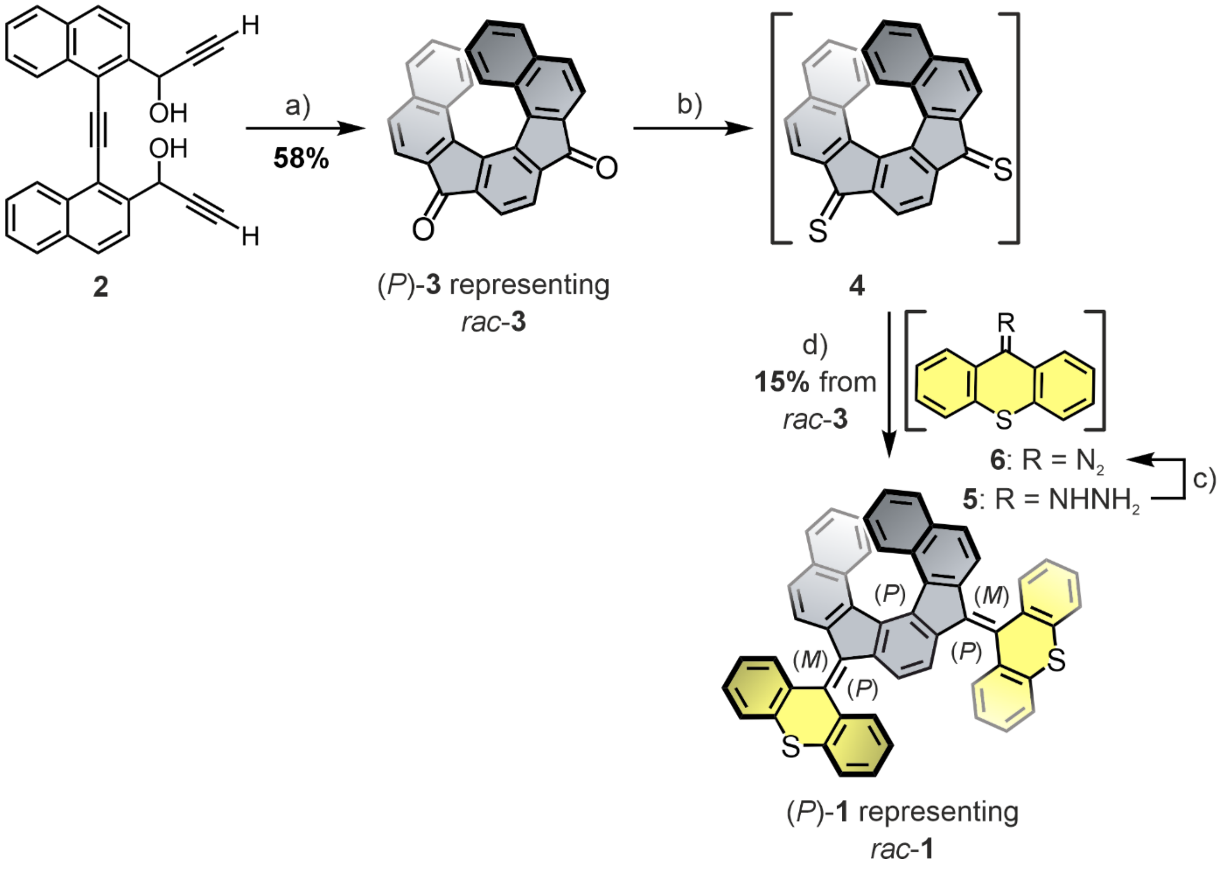

Figure S1. Synthetic scheme towards racemic *rac*-**1**. Conditions: a) 1. Ag_2CO_3, $[RhCl(PPh_3)_3]$, THF, mw, 180 °C, 1.5 h; 2. Pyridinium chlorochromate, celite, CH_2Cl_2, rt, 3 h. b) Lawesson's reagent, toluene, reflux, 1.5 h. c) Ag_2O, KOH (sat. in methanol), $MgSO_4$, diethyl ether, 0 °C, 45 min. d) Hexamethylphosphorous triamide (HMPT), toluene, diethyl ether, rt, 15 min.

Synthesis of *rac*-**3**:

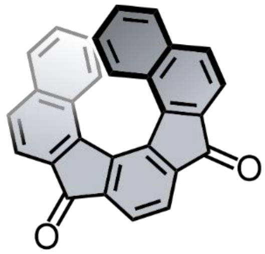

(*P*)-**3** representing *rac*-**3**

The synthesis of *rac*-**3** was adapted from literature in order to obtain a racemic mixture.[3]

A microwave vial was charged with **2**[4] (40 mg, 104 µmol, 1 eq.) followed by the addition of THF (3 mL). The solution was degassed with nitrogen for 5 min before adding a spatula tip of Ag_2CO_3 and $[RhCl(PPh_3)_3]$ and heating the mixture to 180 °C for 1.5 h in a microwave reactor. After cooling the reaction mixture to room temperature, the solvent was evaporated under reduced pressure followed by the addition of CH_2Cl_2 (10 mL), pyridinium chlorochromate (67 mg, 311 µmol, 3 eq.) and celite (120 mg). The reaction mixture was stirred at room temperature for 3 h before filtering it over SiO_2/celite (1:4), followed by washing with CH_2Cl_2 until a colorless elution was obtained. Evaporation of the solvent under reduced pressure yielded pure racemic *rac*-**3** (23 mg, **58%**) as a red solid.

The analytical data of the obtained compound matched with literature reported data.[4]

Synthesis of *rac*-**1**:

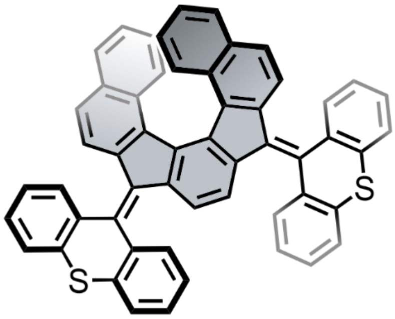

(*P*)-**1** representing
rac-**1**

A flame-dried round-bottom flask was charged with *rac*-**3** (30 mg, 78 µmol, 1 eq.) and Lawesson's reagent (95 mg, 235 µmol, 3 eq.) followed by the addition of dry toluene (12 mL). The flask was equipped with a condenser and the mixture was heated to reflux for 1.5 h. After cooling the reaction mixture to room temperature, the mixture was filtered over a SiO_2 plug into a flame-dried Schlenk flask. The plug was further eluted with toluene until an almost colorless solution eluted (~10 mL). The resulting black solution containing the dithioketone **4** was degassed with nitrogen for 10 min and used without purification for the subsequent step.

In the meantime, a flame-dried microwave vial was charged with **5**[5] (177 mg, 784 µmol, 1 eq.) followed by the addition of dry diethyl ether (10 mL), $MgSO_4$ (640 mg) and a saturated solution of KOH in methanol (1 mL). The mixture was cooled to 0 °C before adding Ag_2O (727 mg, 3.14 mmol, 4 eq.) followed by stirring the reaction mixture vigorously for 1 h at the same temperature. After stopping the stirring, 5 mL of the resulting purple supernatant containing the diazo compound **6** (4.6 eq. wrt. *rac*-**3**) was added to the previously prepared solution of **4**. The resulting reaction mixture was stirred at room temperature for 45 min before adding hexamethylphosphorous triamide (HMPT, 100 µL, 6.6 eq.). After stirring for another 15 min, the mixture was diluted with water and toluene and the phases were separated. The aqueous phase was extracted with toluene and the combined organic layers were washed with brine and dried with $MgSO_4$ before filtration and evaporation of the solvent under reduced pressure. The resulting crude product was purified by automated flash column chromatography (SiO_2, pentane/CH_2Cl_2 1:0 to 8:2 to 0:1) followed by precipitation from a CH_2Cl_2/methanol mixture to yield pure *rac*-**1** (9 mg, **15%**) as an orange solid.

^{1}H NMR (600 MHz, CD_2Cl_2) δ 8.05 – 8.01 (m, 2H), 7.97 (d, *J* = 8.6 Hz, 2H), 7.79 – 7.77 (m, 2H), 7.76 – 7.71 (m, 6H), 7.50 (d, *J* = 8.7 Hz, 2H), 7.46 – 7.40 (m, 4H), 7.37 (d, *J* = 8.7 Hz, 2H), 7.30 – 7.27 (m, 2H), 7.24 – 7.21 (m, 2H), 7.20 – 7.16 (m, 2H), 7.03 (s, 2H), 6.82 – 6.77 (m, 2H); ^{13}C NMR (151 MHz, CD_2Cl_2) δ 143.54, 140.93, 137.84, 137.61, 137.33, 137.26, 137.07, 136.75, 136.69, 134.21, 133.23, 130.14, 129.90, 129.51, 128.93, 128.79, 128.47, 128.03, 127.82, 127.78, 127.09, 126.80, 126.76, 126.13, 125.33, 123.47, 121.01; HR-APCI-MS (+): *m/z* calcd. for $C_{54}H_{31}S_2$ $[M+H]^+$: 743.1862, found: 743.1860.

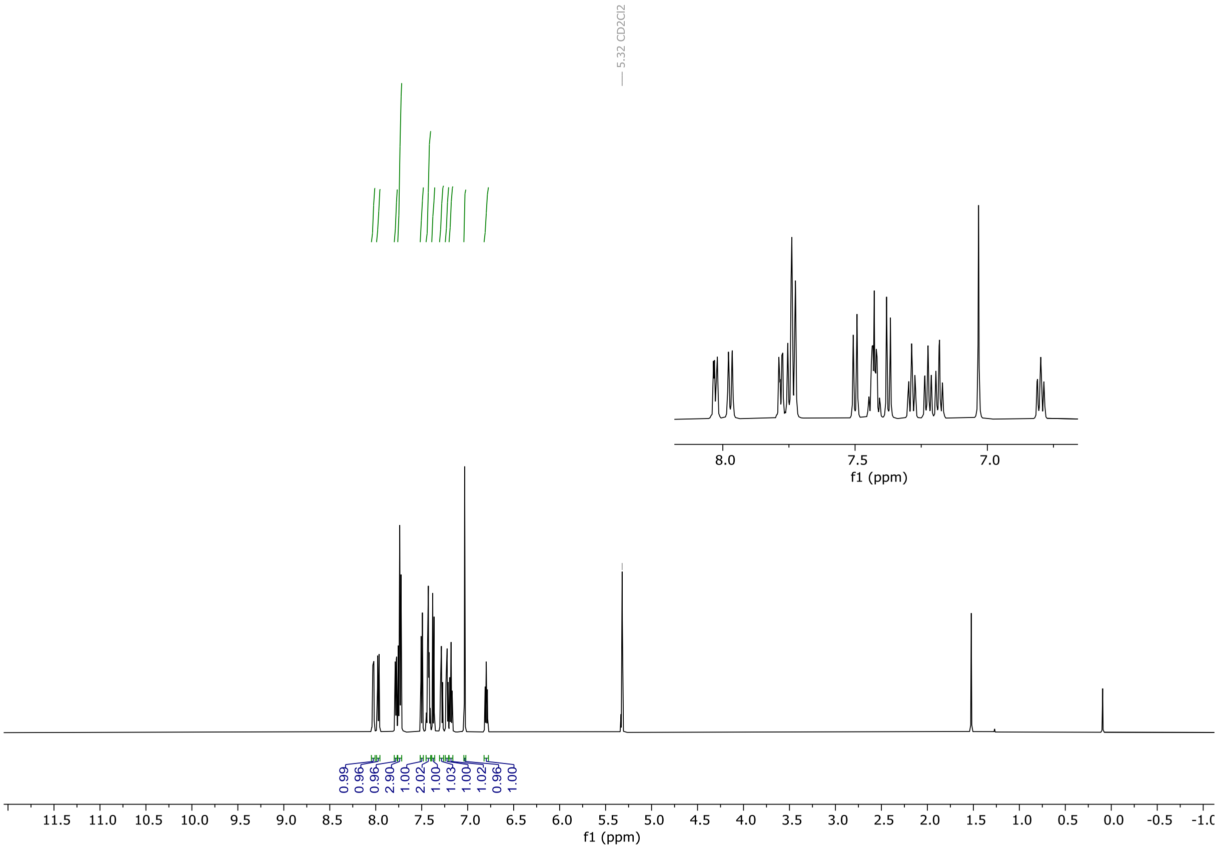

Figure S2. ^{1}H NMR (600 MHz, CD_2Cl_2, 298 K) spectrum of *rac*-**1**.

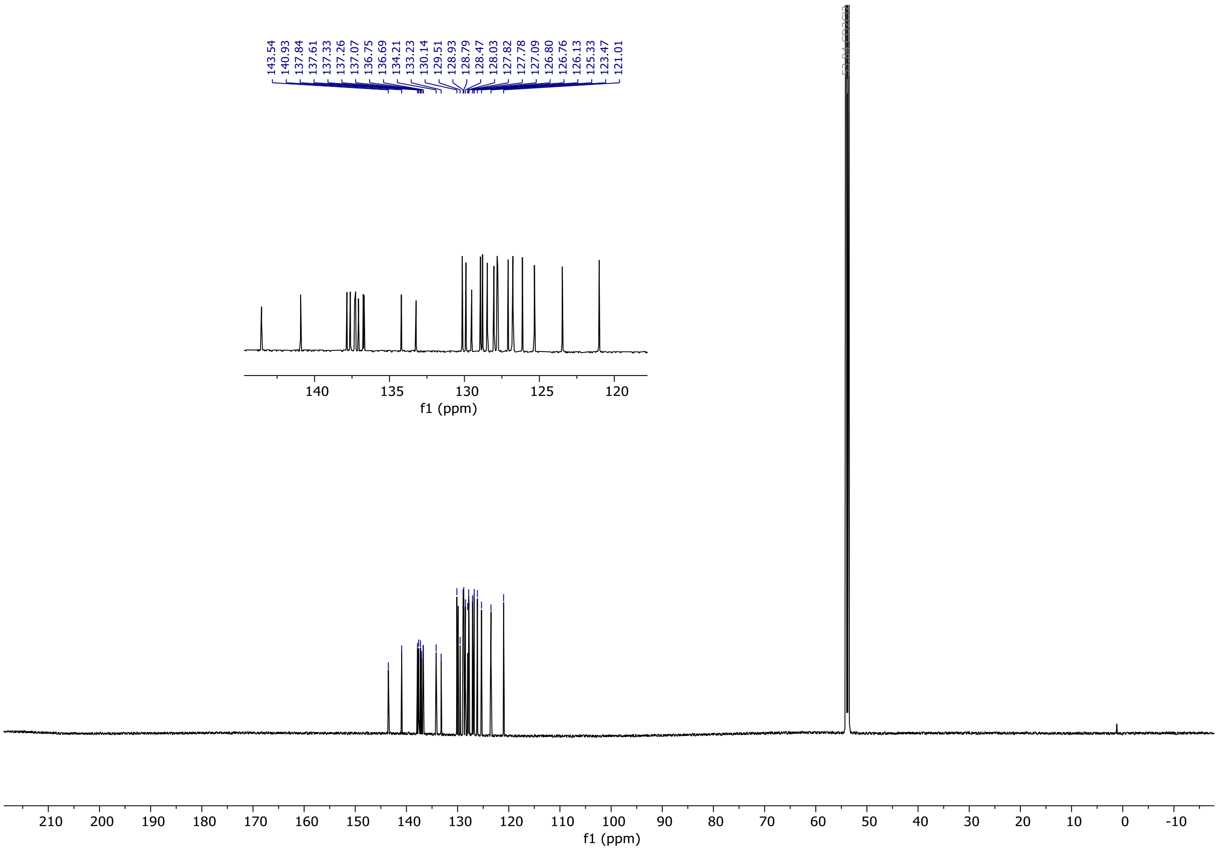

Figure S3. ^{13}C NMR (151 MHz, CD_2Cl_2, 298 K) spectrum of *rac*-**1**.

X-Ray Crystallography (CCDC 2341064)

Switch *rac*-**1** was crystallized by slow diffusion of a layer of methanol on top of a layer of the compound dissolved in CD_2Cl_2. A single-crystal was mounted on a cryoloop and placed in the nitrogen stream (100 K) of a Bruker-AXS D8 Venture diffractometer with a Cu Kα (λ = 1.54178 Å) source. Data collection and processing was carried out using the APEX4 software suite from Bruker.[6] The structure was solved using SHELXT[7] and refinement performed using SHELXL[8] in the OLEX2 software package.[9] The cif file of the structure is provided as additional file. Contributions from disordered solvent were removed using the PLATON/SQUEEZE routine.[10] No A- or B-level alerts were raised by CheckCIF for the fully refined structure of switch *rac*-**1**.

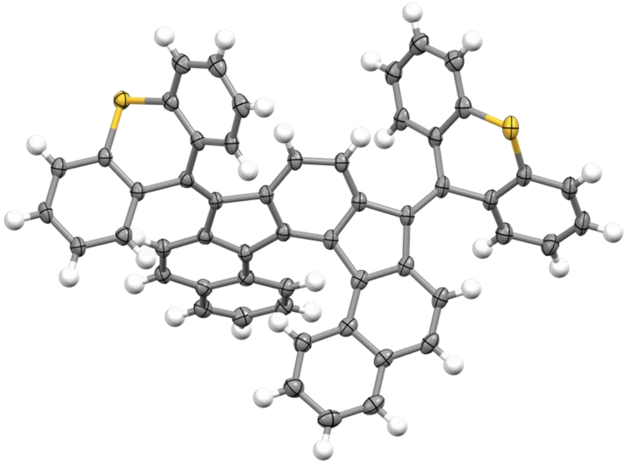

Figure S4. ORTEP plot (ellipsoid at 50% probability) of switch *rac*-**1**.

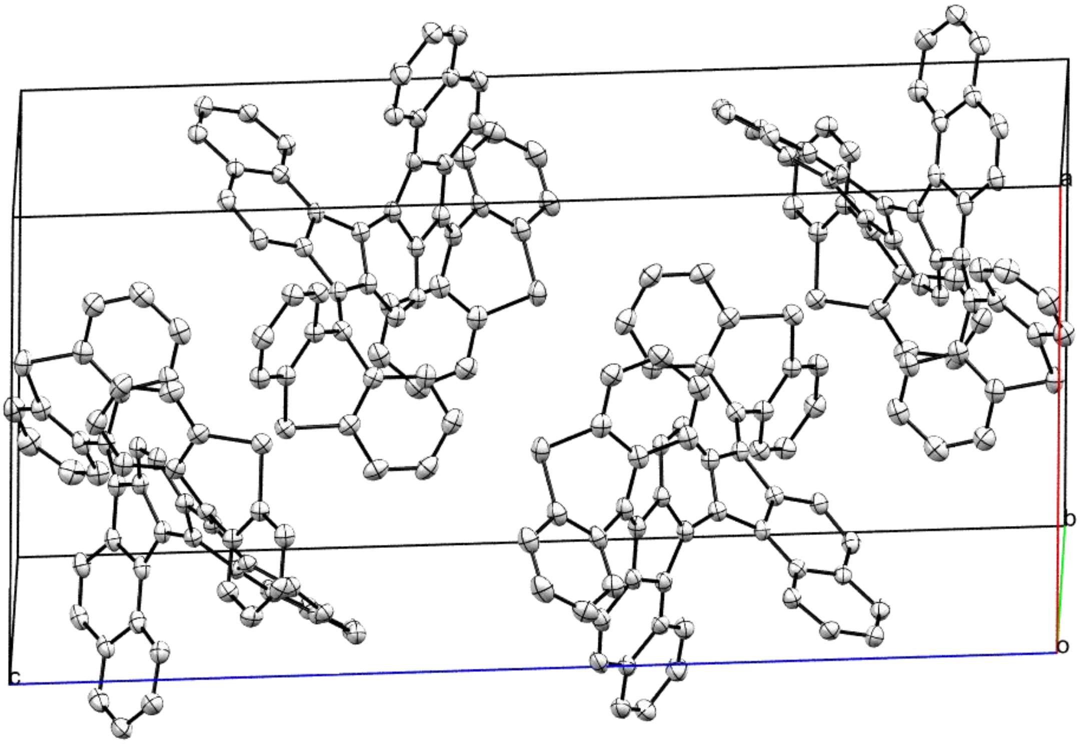

Figure S5. ORTEP plot (ellipsoid at 50% probability) of the unit cell of switch *rac*-**1**. Hydrogens are omitted for clarity.

Table S1. Crystal data and structure refinement for switch *rac*-**1**.

Empirical formula	$C_{54}H_{30}S_2$
Formula weight	742.90
Temperature/K	100.00
Crystal system	monoclinic
Space group	$P2_1/c$
a/Å	12.8590(3)
b/Å	11.1400(2)
c/Å	27.5169(6)
α/°	90
β/°	92.550(2)
γ/°	90
Volume/Å^3	3937.87(14)
Z	4
ρ_{calc}g/cm^3	1.253
μ/mm^{-1}	1.505
F(000)	1544.0
Crystal size/mm^3	0.14 × 0.12 × 0.08
Radiation	CuKα (λ = 1.54184)
2Θ range for data collection/°	6.43 to 141.366
Index ranges	$-15 \le h \le 15, -13 \le k \le 13, -33 \le l \le 33$
Reflections collected	92176
Independent reflections	7550 [R_{int} = 0.1350, R_{sigma} = 0.0528]
Data/restraints/parameters	7550/0/508
Goodness-of-fit on F^2	1.050
Final R indexes [I>=2σ (I)]	R_1 = 0.0639, wR_2 = 0.1602
Final R indexes [all data]	R_1 = 0.0811, wR_2 = 0.1718
Largest diff. peak/hole / e Å^{-3}	0.35/-0.68

UV-Vis Dilution Series

The absorption spectra of a series of diluted samples of *rac*-**1** were measured (Figure S6). The absorption value at 408 nm was then plotted vs. the concentration of the solution and a linear fit was applied (Figure S7). The linear relationship validates the adherence to the Lambert-Beer-Law and thus confirms absence of any aggregation. Furthermore, it enabled exact determination of the concentration of diluted samples when weighing was troublesome (e.g. pure enantiomers, where only minor amounts of samples were at hand).

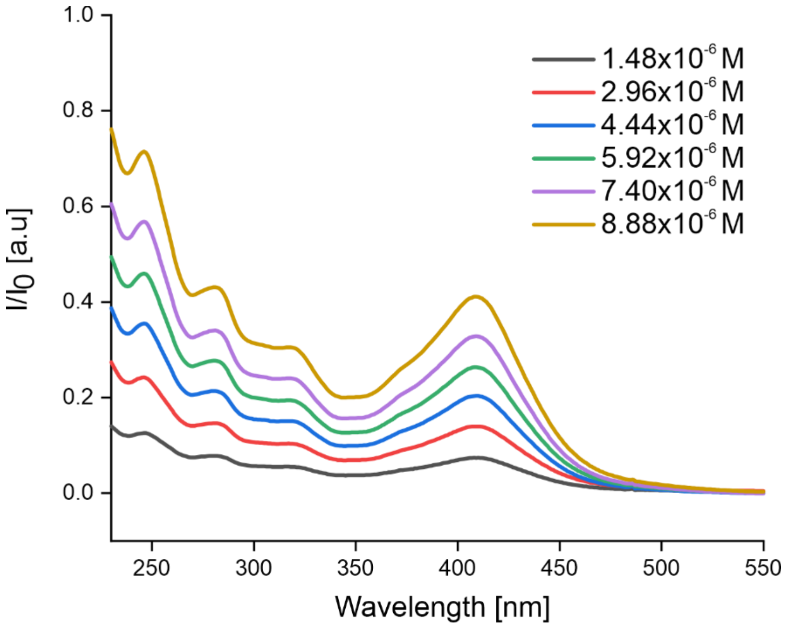

Figure S6. Absorption spectra of solutions of *rac*-**1** in CH_2Cl_2 with different concentrations.

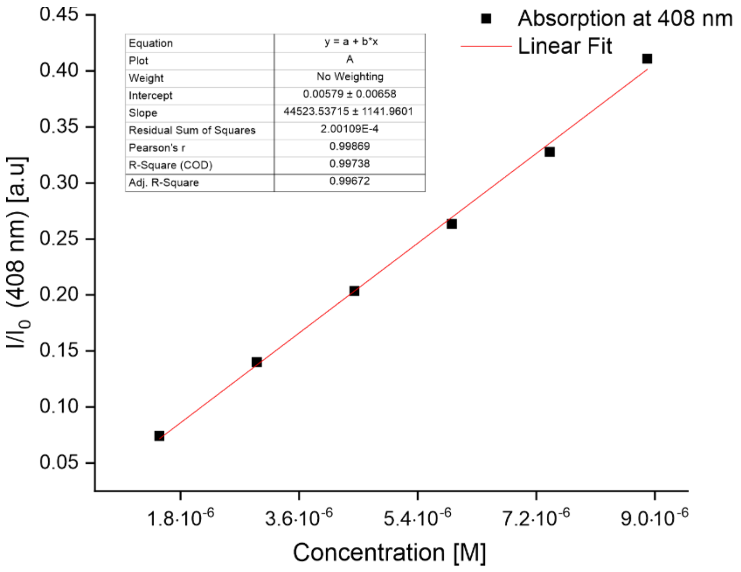

Figure S7. The absorbance at 408 nm of differently concentrated solutions of *rac*-**1** in CH_2Cl_2 is plotted versus its concentration and linearly fitted.

HPLC Separation & g-Factor Plot

Separation of enantiomers of *rac*-**1** was performed using a Shimadzu Prominence HPLC system equipped with a chiral CHIRALPAK IE column from Daicel Corporation and a photo diode array detector.

Analytical separation was achieved by using *n*-heptane/CH_2Cl_2 (7:3) as the mobile phase (flowrate 0.8 mL/min) on a 4.6x250 mm column (5 µm particle size) and injecting 10 µL of the sample (0.8 mg/mL in the mobile phase).

Semipreparative separation was achieved using *n*-heptane/CH_2Cl_2 (7:3) as the mobile phase (flowrate 3.5 mL/min) on a 10x250 mm column (5 µm particle size) and injecting 60 µL of the sample (2.2 mg/mL in the mobile phase).

Assignment of the absolute configuration to the peaks in the chromatogram was based on DFT calculations (*vide infra*).

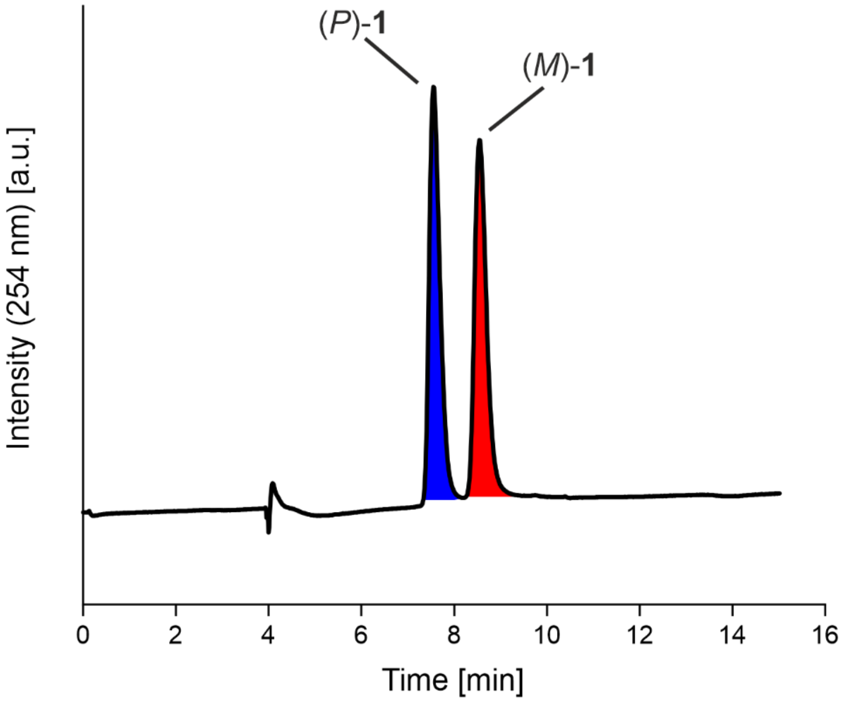

Figure S8. Analytical HPLC chromatogram of *rac*-**1** on a chiral stationary phase.

Enantiomeric excess was determined by reinjecting the isolated enantiomers (*P*)-**1** and (*M*)-**1** to analytical separation conditions and integrating the area beneath the absorption peaks of the chromatogram at 254 nm.

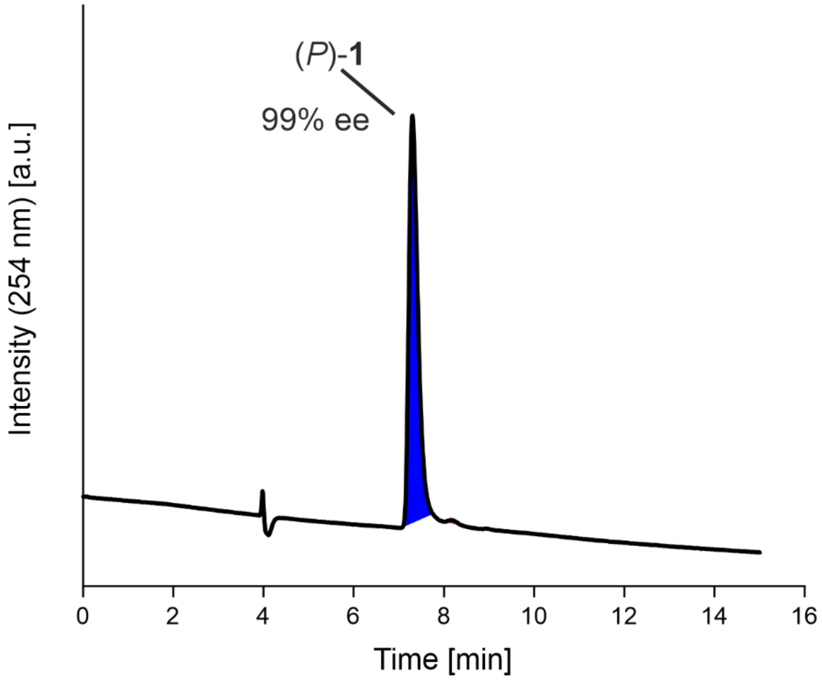

Figure S9. Analytical HPLC chromatogram of (*P*)-**1** after semipreparative separation on a chiral stationary phase.

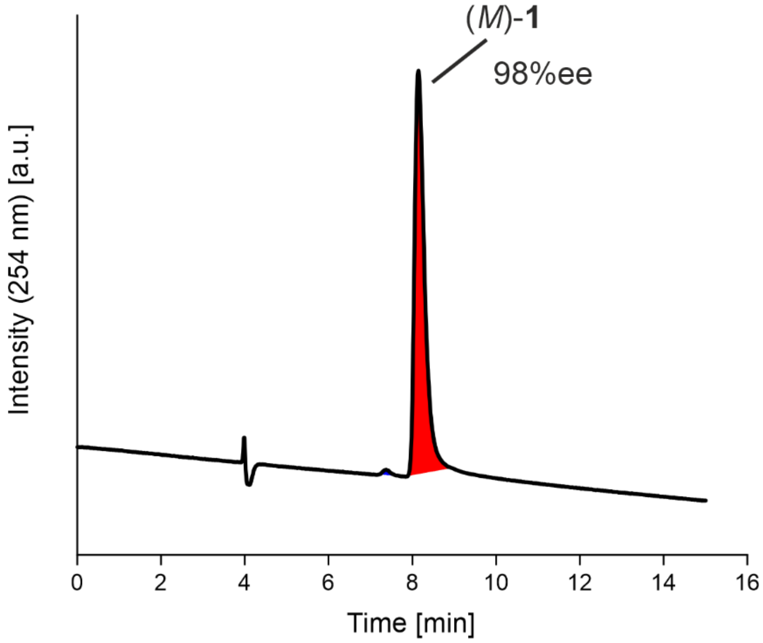

Figure S10. Analytical HPLC chromatogram of (*M*)-**1** after semipreparative separation on a chiral stationary phase.

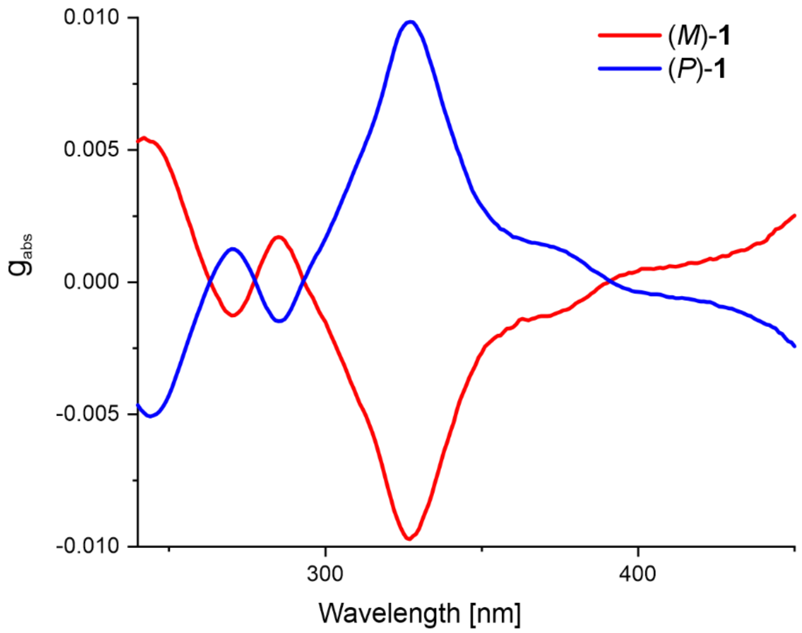

Figure S11. g-Factor plot (Δε/ε) for (*M*)-**1** (red) and (*P*)-**1** (blue).

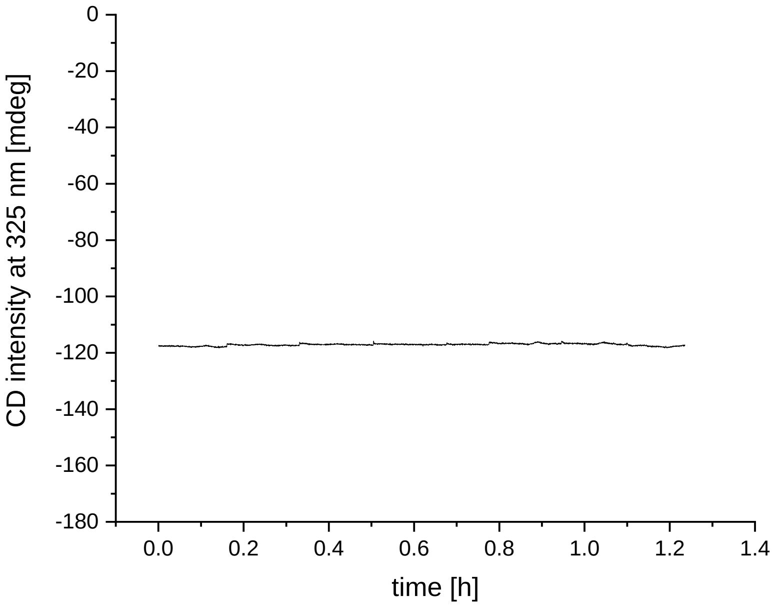

Figure S12. Changes in CD intensity of (*M*)-**1** at 325 nm in toluene at 85 °C over time, showing no racemization at this temperature.

In the case of (*P*)-$\mathbf{1}^{2+}$ (generated by oxidation with 2 equivalents of magic blue), a decay curve of the CD signal at 325 nm was observed at 75 °C in 1,2-dichloroethane. However, the absorption spectrum of the sample at the end of the experiment differed considerably from the initial absorption spectrum, which indicates decomposition/side-reaction rather than a clean racemization process. It should be noted that at room

temperature no racemization was observed during the time course of the spectroelectrochemical measurements, indicating that the dication is also configurationally stable.

^{1}H NMR Chemical Redox Studies

To a solution of *rac*-**1** in CD_2Cl_2 (≈0.5 mg/mL) was added an excess of solid $Fe(ClO_4)_3{\cdot}xH_2O$ (~20 mg). The resulting suspension was briefly sonicated whereupon its colour changed to dark purple. The solid oxidant was then removed by filtration through a syringe filter (0.45 µm) and the NMR spectrum of the so-generated $\mathbf{1}^{2+}$ recorded. Re-reduction was performed analogously using solid Zn powder. Sonication was carried out until the original orange colour of the solution was recovered. If the oxidation with $Fe(ClO_4)_3{\cdot}xH_2O$ was carried out for too long, unknown over-oxidized species were obtained.

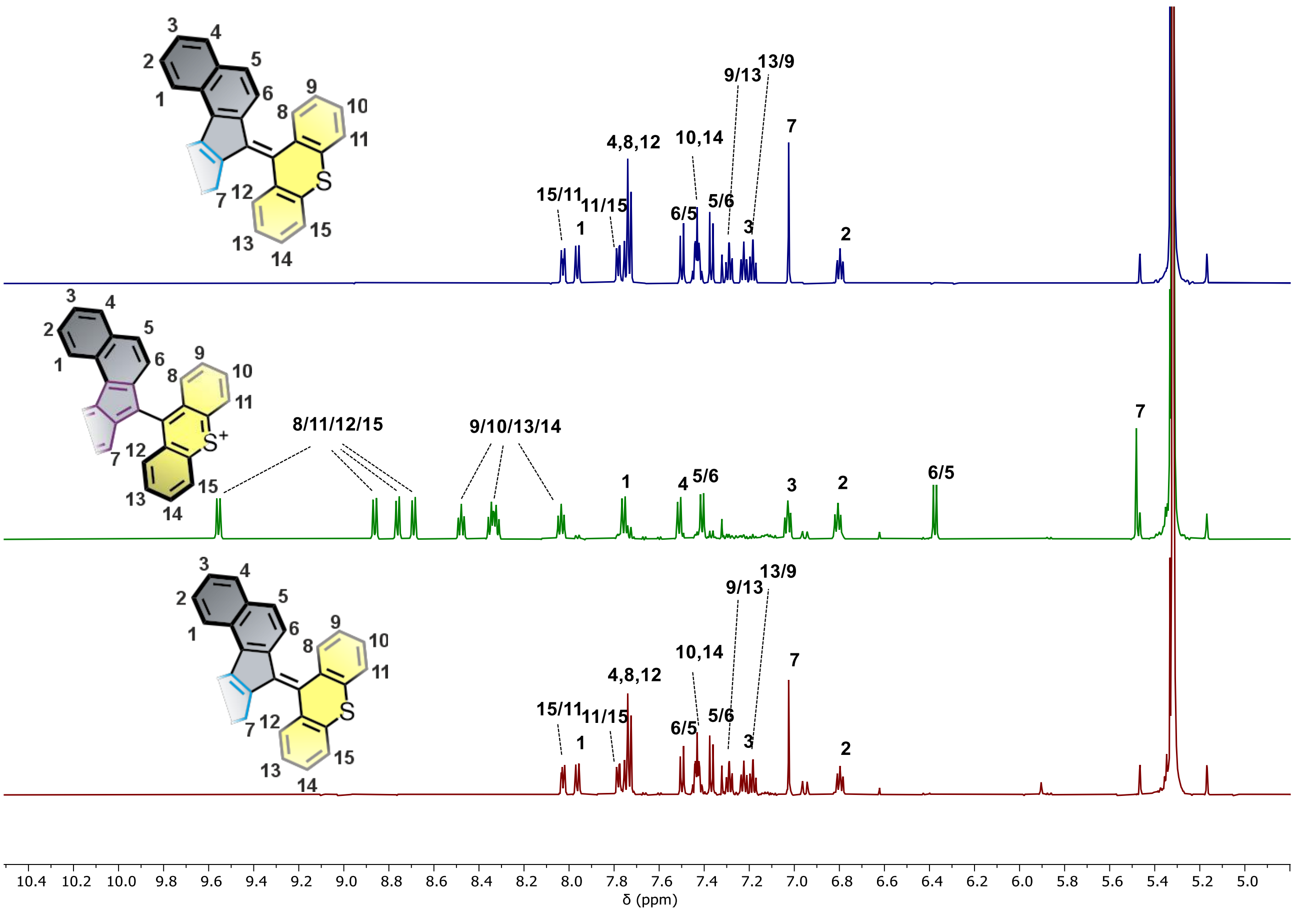

Figure S13. Stacked ^{1}H NMR spectra (CD_2Cl_2, 600 MHz) of **1** (top), $\mathbf{1}^{2+}$ obtained by oxidation with $Fe(ClO_4)_3$ (middle) and re-reduced **1** obtained by reduction with Zn (bottom) in CD_2Cl_2.

Alternatively, chemical oxidation was also performed using trifluoroacetic acid (TFA). A suspension of *rac*-**1** in TFA-d was stirred at room temperature over the course of two months, which slowly oxidized *rac*-**1** to *rac*-**1**$^{+}$, yielding a deeply purple colored solution. A ^{1}H NMR spectrum was recorded after filtering off remaining starting material and adding CD_2Cl_2 as lock reference. In the oxidation with TFA, no over-oxidized species are obtained and the dication *rac*-**1**$^{2+}$ is stable in solution over months.

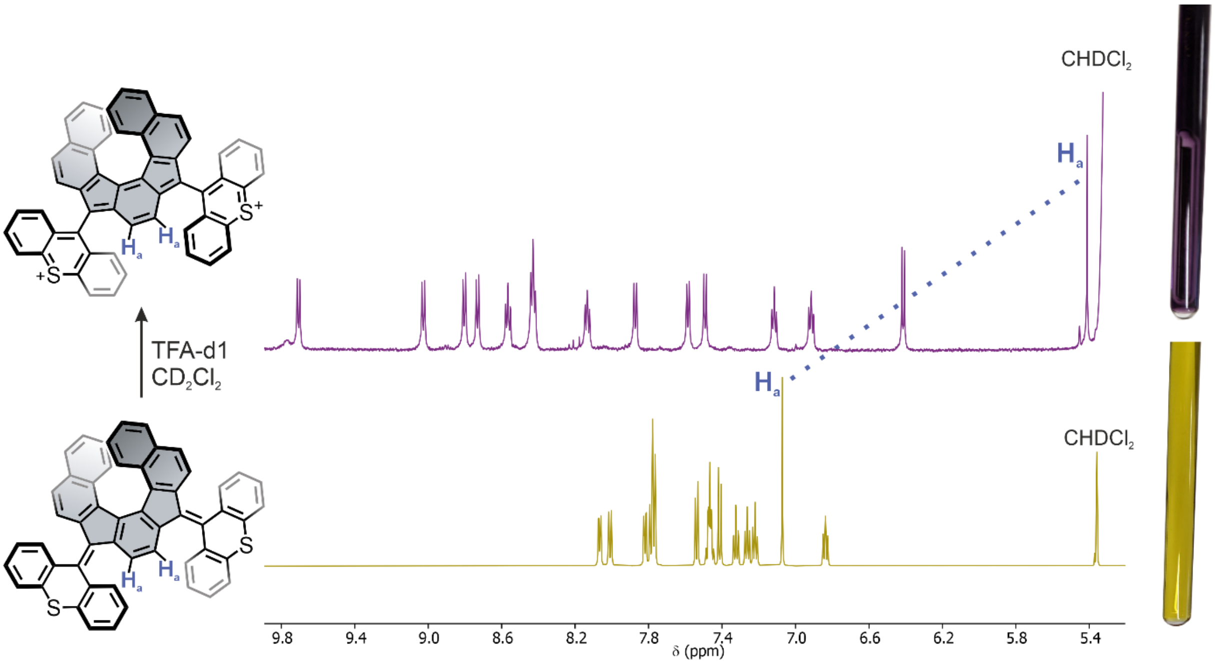

Figure S14. Stacked ^{1}H NMR spectra (CD_2Cl_2, 600 MHz) of *rac*-**1** (bottom) and *rac*-**1**$^{2+}$ (top) obtained by oxidation with TFA-d. The intense colored solutions are displayed on the right-hand side.

To assess the diradical character of (*rac*)-**1**$^{2+}$ (obtained via in situ oxidation with $Fe(ClO_4)_3$), variable-temperature NMR (VT-NMR) in deuterated 1,1,2,2-tetrachloroethane (TCE-d_2) was performed (Figure S15). No considerable line broadening was observed up to a temperature of 80 °C. However, side reactions started to occur at 60 °C, which were considerably accelerated at 90 °C, potentially leading to the observed line broadening of some of the peaks. These results are, as expected for an indeno[2,1-*c*]fluorene scaffold, indicative of a low diradical character of the *as*-indacene.[11]

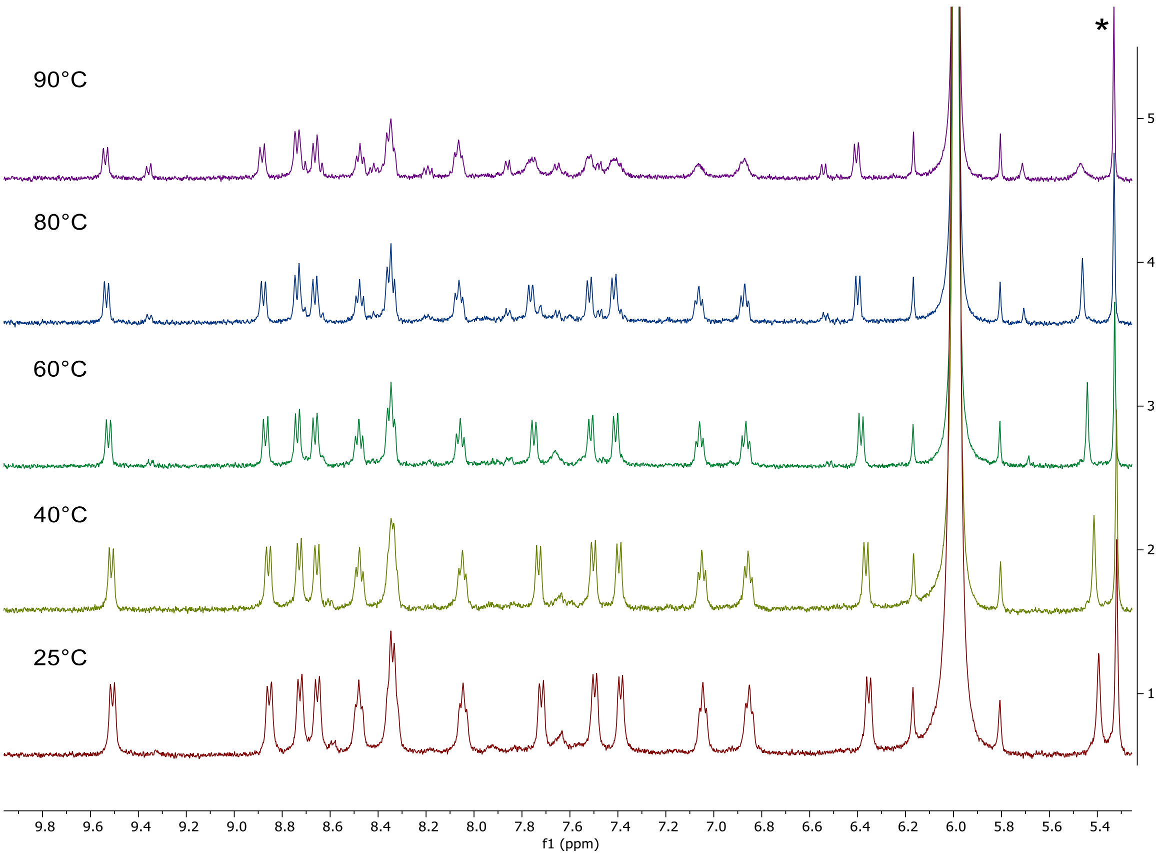

Figure S15. VT-NMR (500 MHz, TCE-d$_2$) of *rac*-**1**$^{2+}$. Residual CH_2Cl_2 is marked with a star.

DFT calculations

Geometry Optimization:

Geometry optimizations of (*P*)-**1** and (*P*)-$\mathbf{1}^{2+}$ were performed with the composite functional r^{2}SCAN-3c/CPCM(CH_2Cl_2)[12,13], using MM2 models as starting points (Figure S16). Potential energy minima were confirmed by subsequent frequency calculations. DFT calculations using the r^{2}SCAN-3c functional have proven to be reliable and cost-efficient for the geometry optimization of various overcrowded alkenes.

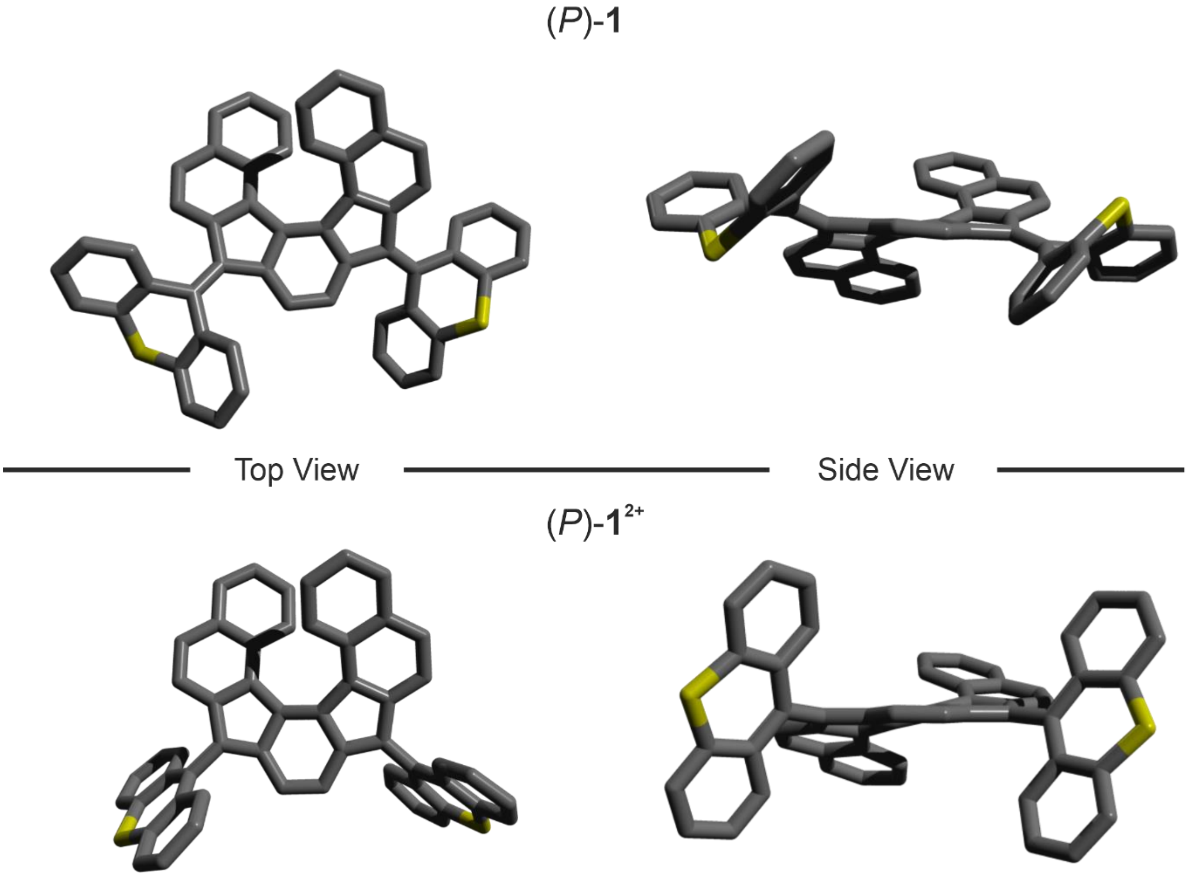

Figure S16. Top and side view of the DFT optimized geometries of (*P*)-**1** (top) and (*P*)-$\mathbf{1}^{2+}$ (bottom). The calculations were performed on a r^{2}SCAN-3c/CPCM(CH_2Cl_2) level of theory.

Further conformational analysis:

A conformational analysis was performed for (*P*)-**1** at the r^{2}SCAN-3c/CPCM(CH_2Cl_2)[12,13] level of theory. Potential energy minima were confirmed by subsequent frequency calculations. Additionally, the transition state from the ground state to the second most stable conformer (TS1) was calculated at the r^{2}SCAN-3c/CPCM(CH_2Cl_2)[12,13] level of theory. The transition state was confirmed by the

presence of a single imaginary harmonic frequency. The side view of the optimized structures as well as their relative energies are displayed in Figure S17.

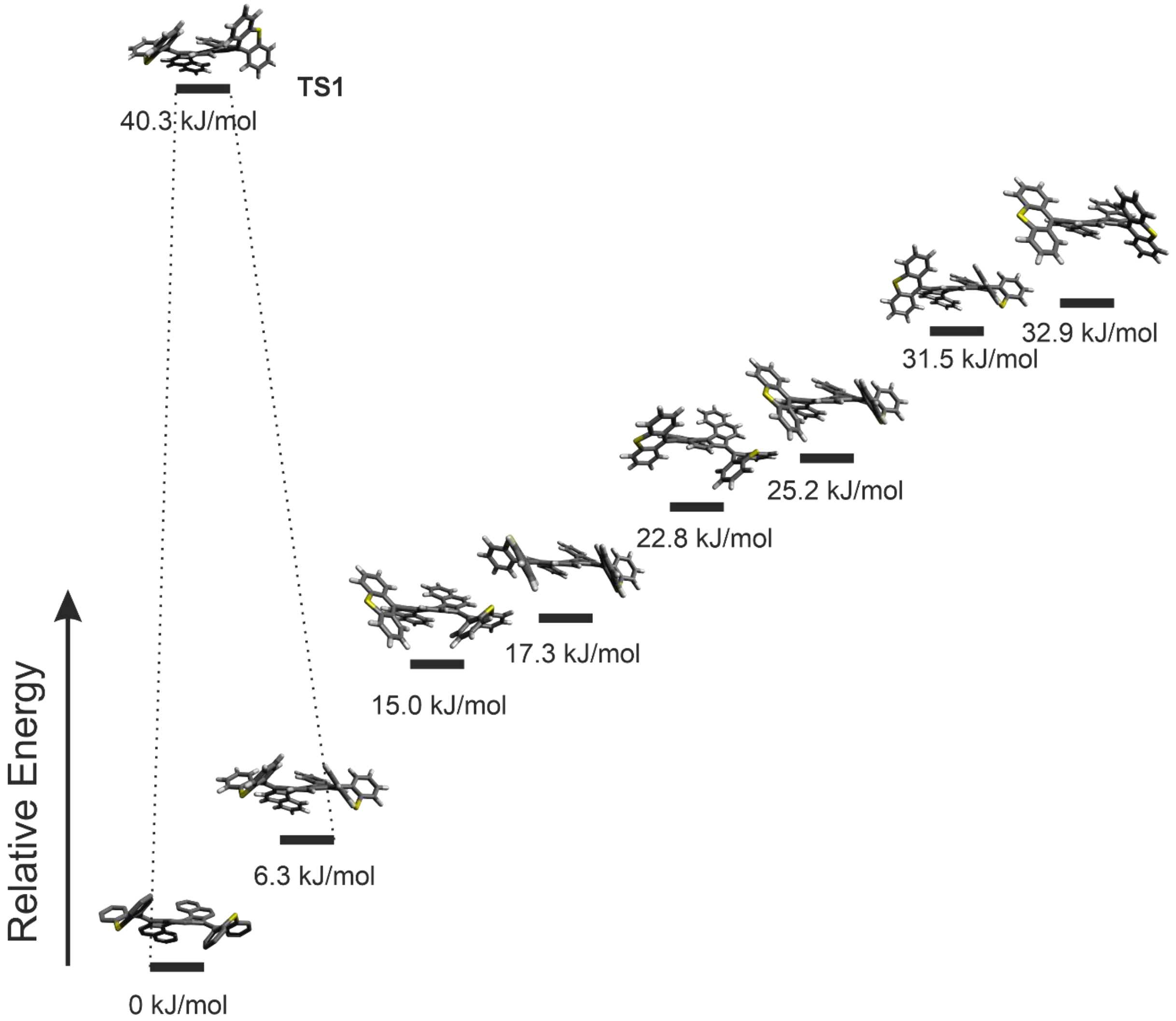

Figure S17. DFT-optimized structures of different conformers and the first transition state (TS1) of (*P*)-**1** from the first to the second most stable conformer at the r²SCAN-3c/CPCM(CH_2Cl_2) level of theory.

<u>NICS-XY scan:</u>

A NICS$_{\pi ZZ}$-XY scan was conducted on (*P*)-**1** and (*P*)-**1**$^{2+}$, using B3LYP/6-311+G* NMR=GIAO level of theory and employing the Aroma package.[14–17] Ghost atoms were placed 1.7 Å above the rings with an inter-ghost-atom distance of 0.1 Å. Considering the symmetry of **1**, only half of the molecule was scanned in both (*P*)-**1** and (*P*)-**1**$^{2+}$.

Full TD-DFT:

Using the optimized structures, full TD-DFT calculations of (*P*)-**1** and (*P*)-**1**$^{2+}$ were performed at the B3LYP/6-311g*/CPCM(CH_2Cl_2)[13,18–20] level of theory and considering 100 singlet transitions. The calculated CD spectrum of (*P*)-**1** (with an energy shift of -83 eV) matches well with the measured spectrum (Figure S18).

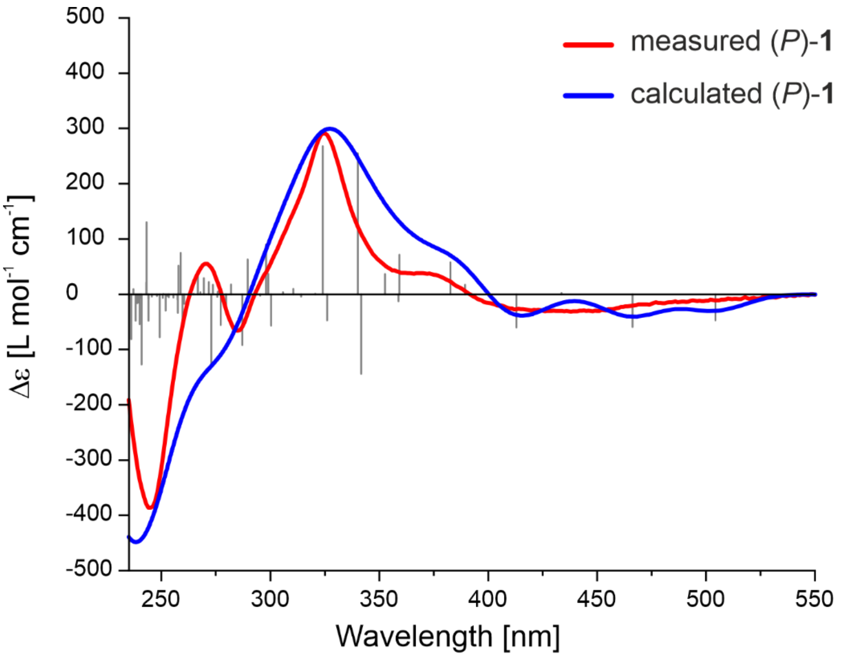

Figure S18. Measured (red) and calculated (blue) CD spectra of (*P*)-**1** at the B3LYP/6-311g*/CPCM(CH_2Cl_2) level of theory. The calculated transitions are plotted as grey lines. The experimental spectrum was measured in CH_2Cl_2 (c~10^{-6} M).

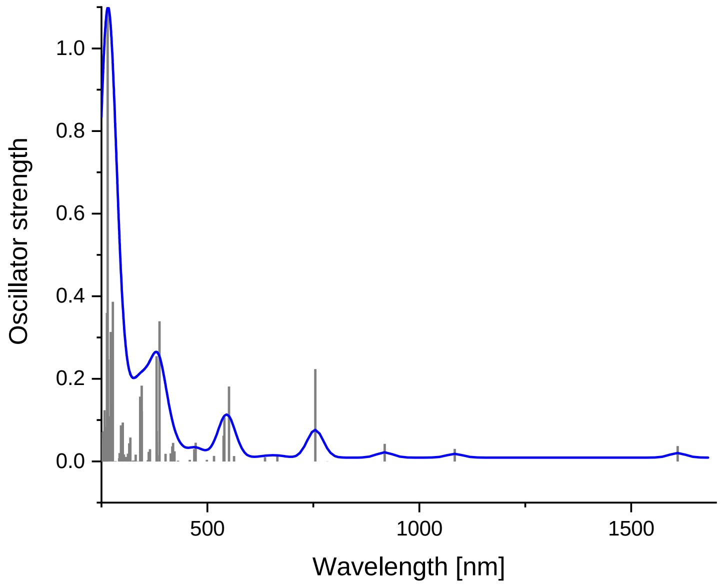

Figure S19. Calculated absorption spectrum of (*P*)-**1**$^{2+}$ (blue line) and the calculated transitions (grey lines) at the B3LYP/6-311g*/CPCM(CH_2Cl_2) level of theory.

To get an understanding of the involved molecular orbitals in the redshifted region of the measured spectrum of **1**$^{2+}$, the full TD-DFT calculations were analyzed. The first calculated 8 states are summarized in Table S2 and the involved molecular orbitals (HOMO-2 to LUMO+2) are plotted in Figure S20. The visualization of the molecular orbitals reveals occupied π orbitals on the central helicene core and unoccupied π* orbitals on the thioxanthylium rotors and the *as*-indacene core. Thus, the redshifted region is partially governed by charge-transfer characteristics from the central helicene core to the cationic thioxanthylium rotors.

Table S2. Summary of the calculated first 8 states of (*P*)-**1**^{2}. The orbitals with the largest individual weight within the respective transition states are written in bold.

State	Energy (eV, nm)	Orbitals	Individual weight	Oscillator strength
1	0.770, 1609	**HOMO → LUMO**<br>HOMO → LUMO+2	**0.966868**<br>0.025855	0.03704
2	1.144, 1084	**HOMO → LUMO+1**	**0.988355**	0.0303213
3	1.350, 918	HOMO-1 → LUMO<br>HOMO → LUMO<br>**HOMO → LUMO+2**	0.021485<br>0.025924<br>**0.938320**	0.0424628
4	1.584, 783	**HOMO-2 → LUMO**<br>HOMO-2 → LUMO+2	**0.967459**<br>0.020770	0.000122991
5	1.643, 754	**HOMO-1 → LUMO**<br>HOMO-1 → LUMO+2<br>HOMO → LUMO+2	**0.952499**<br>0.011087<br>0.022636	0.223565
6	1.865, 664	**HOMO-1 → LUMO+1**	**0.981551**	0.0149556
7	1.950, 636	**HOMO-2 → LUMO+1**<br>HOMO-1 → LUMO+2	**0.969073**<br>0.016550	0.0102897
8	2.203, 563	HOMO-2 → LUMO<br>**HOMO-2 → LUMO+2**	0.021223<br>**0.957449**	0.0129797

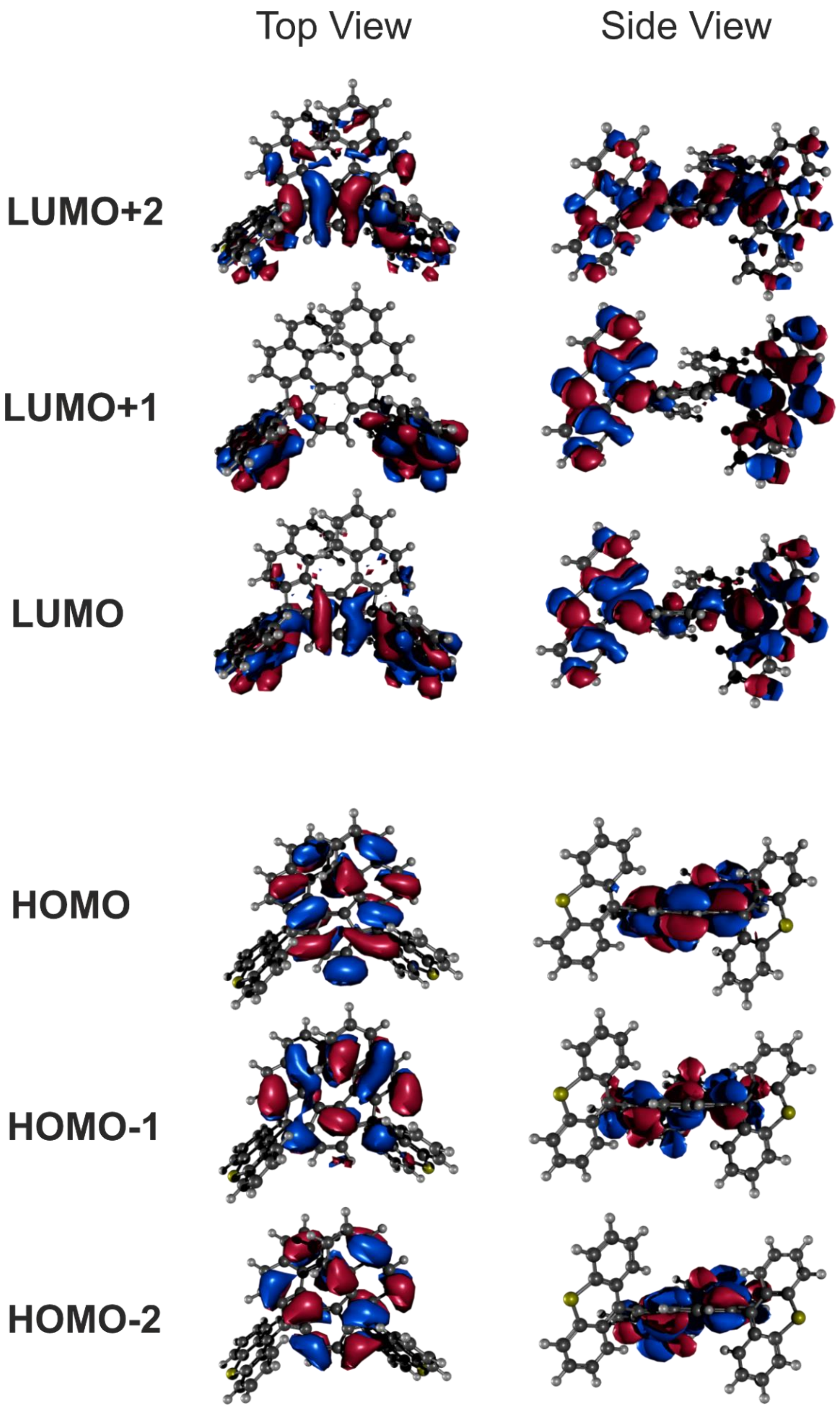

Figure S20. Top and side view of HOMO-2 to LUMO+2 of (*P*)-**1**$^{2+}$ at the B3LYP/6-311g*/CPCM(CH_2Cl_2) level of theory.

Electrochemical Measurements and DYREX Mechanism

Prior to each measurement the GC working electrode was mechanically polished with 0.05 µm alumina slurry and briefly sonicated in H_2O/EtOH (1:1). $TBAPF_6$ was used as electrolyte in all cases (100 mM for standard voltammetric experiments and 200 mM for all spectroelectrochemical experiments). All measurements were performed in DCM.

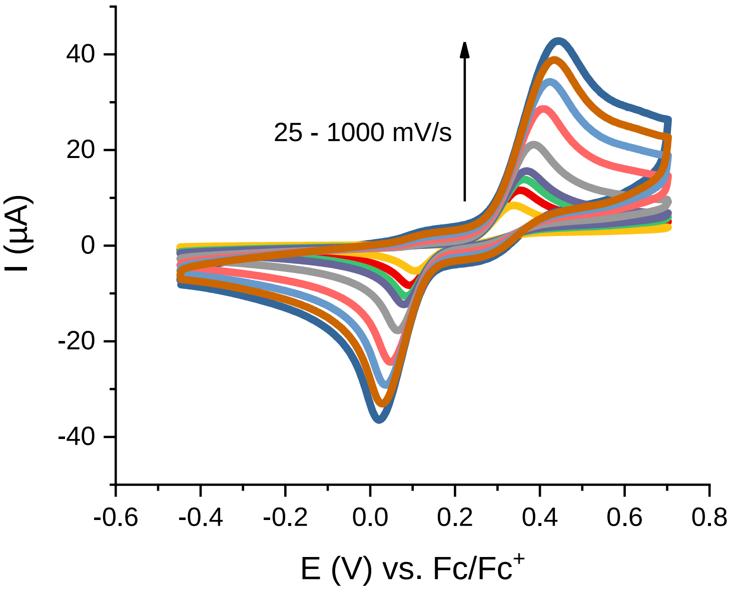

Figure S21. CVs of 0.5 mM **1** in CH_2Cl_2, 100 mM $TBAPF_6$ at varying scan rates.

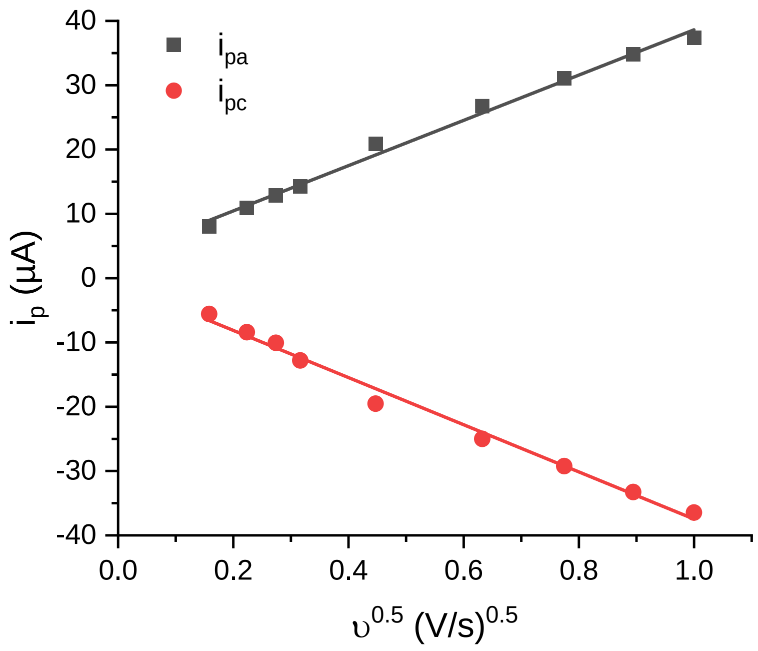

Figure S22. Anodic and cathodic peak currents of 0.5 mM **1** in CH_2Cl_2, 100 mM $TBAPF_6$ as a function of the square-root of the scan rate including linear fits.

As shown in Figure S23, we initially hypothesized that the one-electron oxidation of the initially neutral $\mathbf{1}_{\mathbf{af,af}}$, wherein both rotors are in the anti-folded state, first transiently generates the radical cation with the same geometric arrangement ($\mathbf{1}^{+\bullet}_{\mathbf{af,af}}$, $E_{Ox}{}^{1}$). This oxidation is associated with conversion of the C-C double bond to a single bond of the oxidized thioxanthylium motif, inducing immediate geometric rearrangement of this rotor to the more favorable twisted state ($\mathbf{1}^{+\bullet}_{\mathbf{tw,af}}$, k_1). For this state, various mesomeric structures can be considered, including the one shown in Figure S23, wherein the radical resides on the second rotor and the fully conjugated antiaromatic *as*-indacene is formed. In analogy to the first step, this species can quickly geometrically rearrange to the "doubly twisted" state ($\mathbf{1}^{+\bullet}_{\mathbf{tw,tw}}$, k_2). One-electron oxidation of this species to the target dication $\mathbf{1}^{2+}_{\mathbf{tw,tw}}$ ($E_{Ox}{}^{2}$) is at least as facile, or presumably even more facile than the initial oxidation ($E_{Ox}{}^{2} \leq E_{Ox}{}^{1}$, potential inversion/compression) such that in effect both oxidations happen simultaneously.

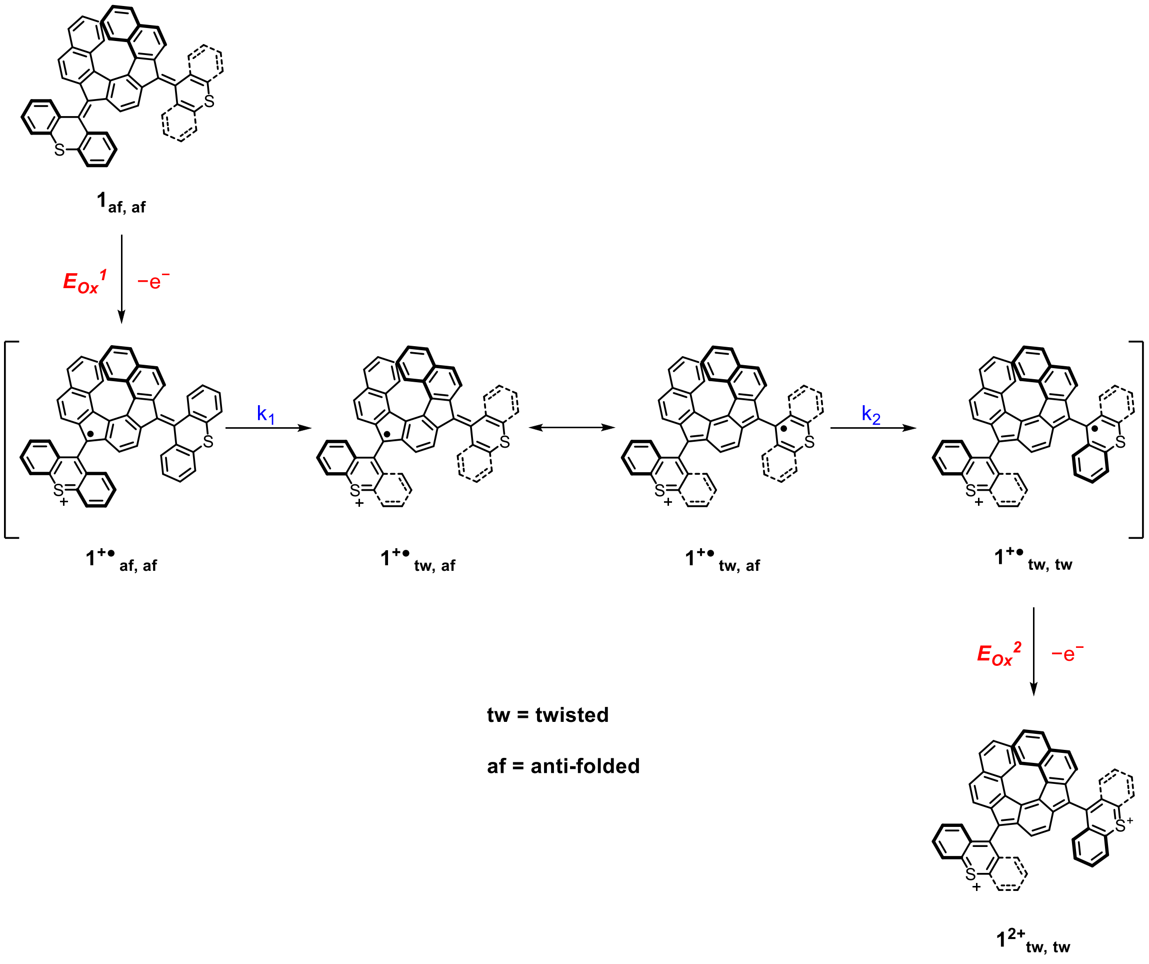

Figure S23. Proposed ECE pathway for oxidation of **1** to **1**$^{2+}$. Red pathways show oxidations, blue pathways geometric rearrangements.

In an alternative mechanism, a conformational rearrangement precedes electron transfer.[21,22] To gain further insight into which mechanism is most likely operating in this system further studies were conducted as detailed in the following.

Low temperature electrochemical studies of **1** were carried out under argon atmosphere by immersion of the electrochemical cell into a dry ice/acetonitrile bath (~–40 °C). During cooling, CVs were continuously recorded until the sample was equilibrated and no further changes were observed. The CV under these conditions (~–40 °C) is shown as the black line in Figure S24, which is qualitatively similar to that obtained at room temperature, albeit with much smaller currents and a larger peak separation. Further cooling to ~–75 °C with a dry ice/acetone bath did not significantly alter the general shape of the CVs.

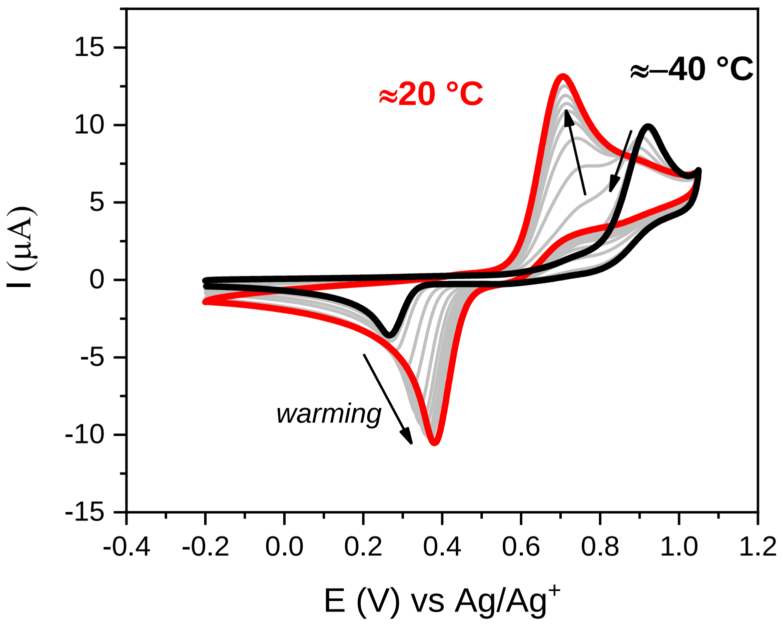

Figure S24. CVs of 0.5 mM **1** in CH_2Cl_2, 100 mM $TBAPF_6$ at v = 100 mV/s at different temperatures between ~–40 °C (black line) and room temperature (red line). The black arrows indicate the changes in the peaks upon warming.

Both the smaller currents and larger peak separation arise, at least in part from the significant change in temperature, i.e. significantly slowed down diffusion and significant IR drop. However, upon removal of the cooling bath and slow warming to room temperature, continuous CV scans showed significant changes that cannot be solely attributed to these effects, in particular for the oxidation wave. Specifically, it is clearly observable that the oxidation peak observed at low temperature disappears upon warming and gives rise to a new (i.e. the "original", room temperature) oxidation wave at lower potentials. This behavior can, for example, arise from a thermal equilibration between different (conformational) species.[21,22] Specifically, the more cathodic oxidation observed at room temperature might arise from oxidation of a minor species of a different geometry with a lower oxidation potential (for example containing twisted rotors). If both the energy difference and the activation energy barrier between this species and the most stable doubly *anti*-folded state is sufficiently low for them to quickly equilibrate at a given temperature, then only oxidation of the conformer with a lower oxidation potential is observed. At lower temperature the population of this higher energy, lower oxidation potential species would be prevented, such that the CVs would only reflect the redox properties of the lowest energy conformer. We attempted to further investigate this by fast-scan CV studies with scan rates up to 20 V/s

(Figure S25). At higher scan rates the oxidation peak appears to broaden, which is potentially the result of appearance of a new peak at higher potential (analogous to the experiments at low temperature and reflective of an equilibrium between different species). However, due to significant distortions at these high scan rates this cannot be clearly resolved.

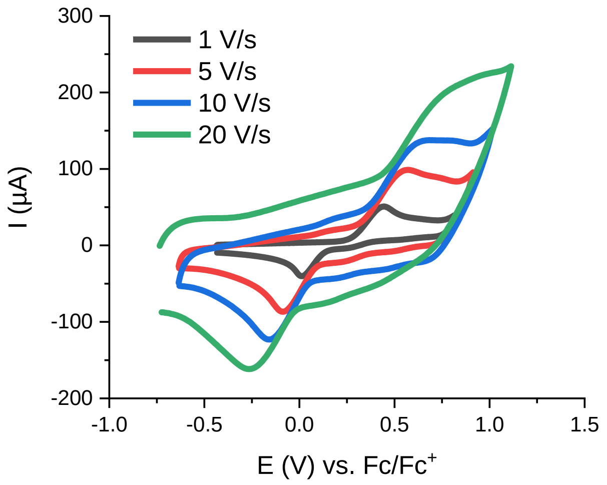

Figure S25. CVs of 0.5 mM **1** in CH_2Cl_2, 100 mM $TBAPF_6$ at high scan rates up to 20 V/s.

To further probe whether different conformers of **1** are accessible, we subjected *rac*-**1** to VT-NMR in TCE-d_2 over a wide temperature range between –28 °C to 80 °C (Figure S26). Across this whole temperature range only small shift differences in the proton signals were observed, which are unlikely to arise from (significant) geometric changes. This was also corroborated by computational conformational analysis, which revealed that numerous other conformers of neutral **1** can in principle exist, whereby one or two of the rotors adopt differently folded or twisted structures (Figure S17). However, these species are all either much higher in energy or thermally inaccessible due to high activation energy barriers, which would suggest that conformational rearrangements do not precede electron transfer.

Taken together, these results do not allow an unambiguous identification of the specific dynamic redox mechanism. However, regardless of temperature, scan rate and the specific mechanism, it appears that all oxidations and reductions occur via virtually simultaneous 2-electron transfers, such that in no cases (the build-up of) the intermediate monoradical cation is observable, as also clearly evidenced by the spectroelectrochemical studies (Figure 7).

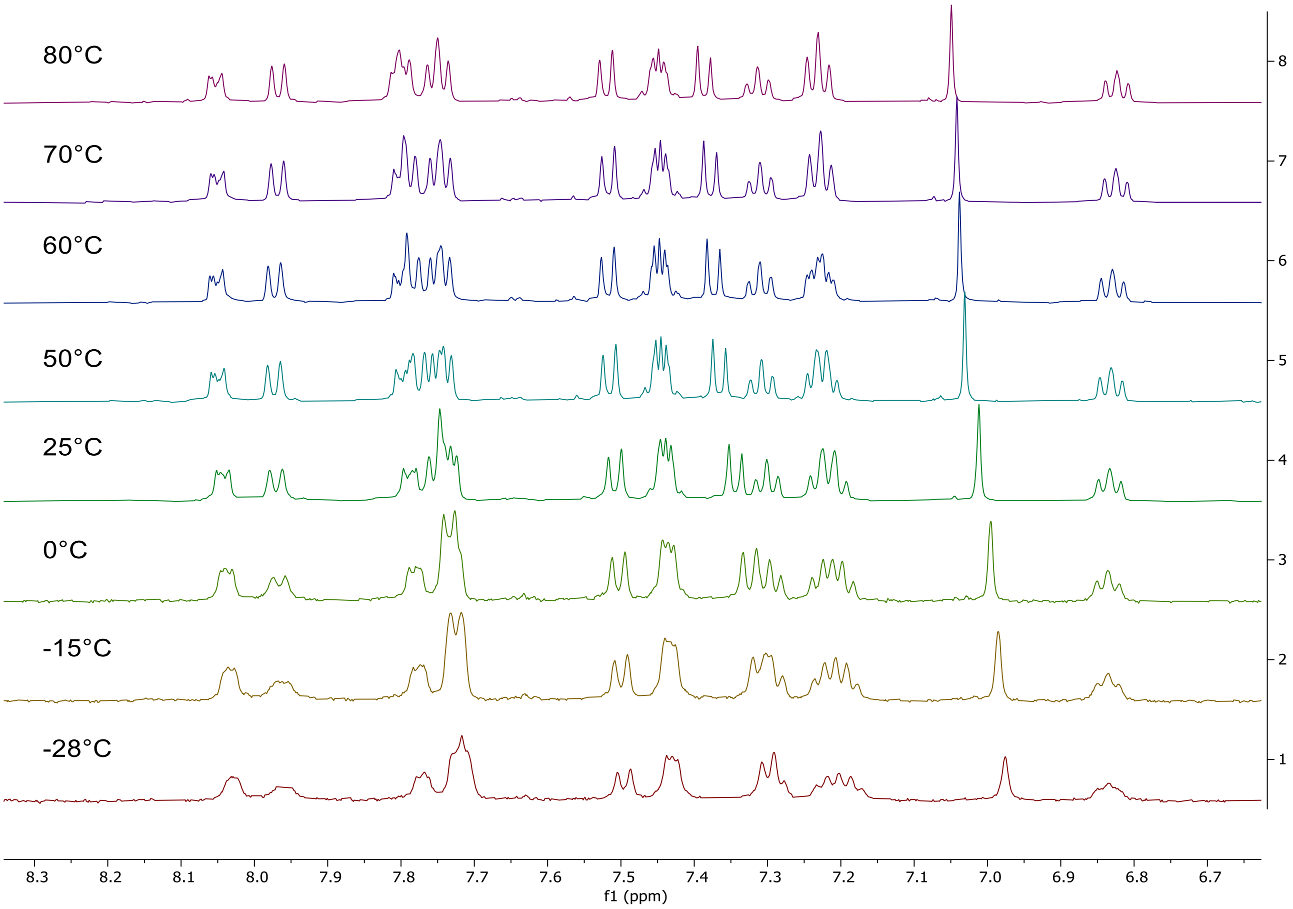

Figure S26. VT-NMR (500 MHz, TCE-d_2) of *rac*-**1**.

Spectroelectrochemistry

A Pt mesh was used as a working electrode in all cases. For oxidation and reduction potentials of +0.70 V or –0.45 V were applied, respectively, typically for 10-20 min per cycle (until full conversion was observed).

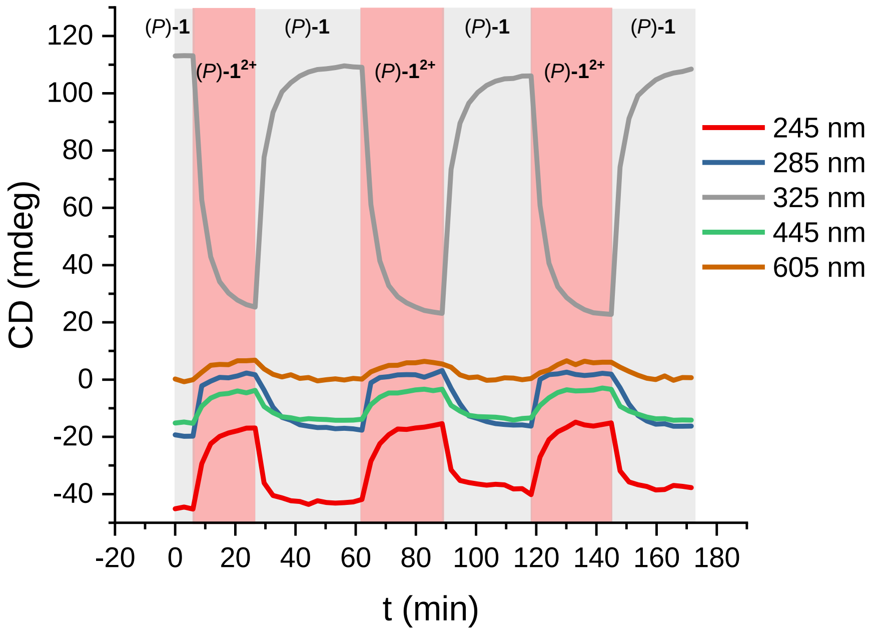

2

Figure S27. Changes in CD signal intensity of (*P*)-**1** in CH_2Cl_2, 200 mM $TBAPF_6$ during spectroelectrochemical cycling between the two oxidation states.

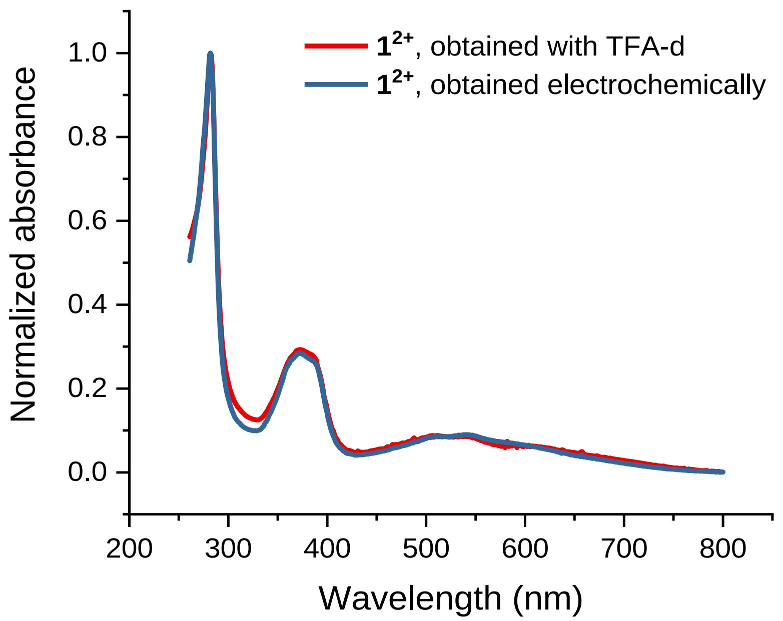

Figure S28. Comparison of normalized absorbance spectra of chemical oxidation of **1** with TFA-d (measured in CH_2Cl_2, red line, see SI section "^{1}H NMR Chemical Redox Studies") and of electrochemical oxidation (measured in CH_2Cl_2, 200 mM $TBAPF_6$, blue line).

Appendix

DFT optimized XYZ coordinates for all conformers in Figure S17 can be found in the supporting .zip file.

<u>The DFT optimized XYZ coordinates of the lowest energy conformers:</u>

(*P*)-**1**:

C	-2.77982	-4.40702	-1.46992
C	-2.55192	-5.79858	-1.49167
C	-1.68805	-6.36430	-0.58422
C	-1.04055	-5.57265	0.39459
C	-1.33854	-4.17177	0.47388
C	-2.19064	-3.61603	-0.51116
C	-0.04929	-6.12805	1.24054
C	0.64288	-5.35098	2.13692
C	0.31086	-3.98923	2.28381
C	-0.69815	-3.41321	1.49508
C	0.97569	-2.92858	3.06906
C	0.43850	-1.66185	2.52371
C	-0.71889	-1.97115	1.75197

C	0.90358	-0.35394	2.60181
C	0.22779	0.66520	1.92920
C	-0.98475	0.39656	1.30389
C	-1.54664	-0.91199	1.35069
C	-1.93590	1.27772	0.59037
C	-3.20338	0.51886	0.56018
C	-2.96821	-0.78778	1.01859
C	-4.51618	0.95786	0.29588
C	-5.56947	0.09087	0.45462
C	-5.37707	-1.21881	0.95881
C	-4.05721	-1.65885	1.30810
C	-6.47889	-2.07496	1.19786
C	-6.30642	-3.29395	1.80943
C	-5.02074	-3.69143	2.23115
C	-3.92597	-2.89607	1.98449
C	-1.68035	2.49224	0.02787
C	-0.39626	3.21637	0.13667
C	-0.41405	4.54232	0.59812
S	-1.97480	5.33440	0.94411
C	-2.91329	4.60064	-0.38314
C	-2.66766	3.26773	-0.74939
C	0.82185	2.67332	-0.28748
C	2.00166	3.39234	-0.15460
C	1.98022	4.67412	0.39729
C	0.77241	5.25918	0.75848
C	-3.87056	5.36038	-1.05615
C	-4.54564	4.80867	-2.13897
C	-4.24605	3.51517	-2.56918
C	-3.31120	2.75401	-1.88104
C	1.83657	-3.08447	4.11381
C	2.24441	-4.39427	4.65972
C	3.61461	-4.67251	4.78796
S	4.80946	-3.41698	4.36736
C	3.89661	-2.00759	4.96952
C	2.49976	-1.97466	4.83045
C	1.32241	-5.33859	5.12561
C	1.75187	-6.56451	5.61465
C	3.11495	-6.86189	5.65278
C	4.04992	-5.91302	5.25503

C	4.57660	-0.96558	5.60112
C	3.85651	0.08713	6.15318
C	2.46151	0.08309	6.10730
C	1.79099	-0.94260	5.45580
H	-3.42391	-3.95419	-2.21857
H	-3.03729	-6.41506	-2.24304
H	-1.46848	-7.42874	-0.61927
H	-2.36917	-2.54823	-0.52207
H	0.19744	-7.18116	1.13037
H	1.45327	-5.78939	2.70466
H	1.81310	-0.11288	3.13655
H	0.63880	1.66614	1.94914
H	-4.71070	1.97801	-0.00833
H	-6.58293	0.42298	0.24343
H	-7.47119	-1.73653	0.90929
H	-7.16067	-3.93895	1.99489
H	-4.89296	-4.63260	2.75862
H	-2.95072	-3.21512	2.32972
H	0.83411	1.66971	-0.70283
H	2.94037	2.95377	-0.47992
H	2.90245	5.23643	0.51065
H	0.74398	6.27890	1.13182
H	-4.06258	6.38522	-0.75137
H	-5.28201	5.40479	-2.67000
H	-4.74477	3.10054	-3.44018
H	-3.08443	1.74009	-2.19896
H	0.26238	-5.10541	5.07621
H	1.02476	-7.29134	5.96427
H	3.45472	-7.82296	6.02756
H	5.11326	-6.12090	5.33345
H	5.65902	-0.99748	5.68708
H	4.38526	0.89226	6.65492
H	1.89751	0.88204	6.57942
H	0.70600	-0.94413	5.40475

(*P*)-**1**$^{2+}$:

C	-0.64222	-2.22039	-4.20995
C	-1.20022	-2.13732	-5.50622

C	-2.41233	-1.52082	-5.69587
C	-3.11104	-0.94374	-4.60599
C	-2.49473	-0.94554	-3.30425
C	-1.26852	-1.63477	-3.13788
C	-4.41540	-0.42104	-4.77292
C	-5.15404	0.01896	-3.69424
C	-4.55291	0.02215	-2.42744
C	-3.20483	-0.32821	-2.24884
C	-5.13582	0.32153	-1.11988
C	-4.15713	0.16620	-0.15442
C	-2.88522	-0.10864	-0.83701
C	-4.26271	0.21333	1.26546
C	-3.14264	0.02552	2.01905
C	-1.88103	-0.08678	1.36706
C	-1.74033	0.01552	-0.09205
C	-0.62092	-0.26879	1.90642
C	0.36665	-0.15797	0.83448
C	-0.30484	0.10385	-0.37091
C	1.76458	-0.24158	0.89948
C	2.49345	0.02038	-0.24176
C	1.86248	0.45225	-1.43234
C	0.42628	0.54671	-1.49723
C	2.62736	0.85029	-2.55741
C	2.02040	1.38652	-3.66607
C	0.61836	1.56705	-3.68855
C	-0.15932	1.15286	-2.63585
C	-0.30364	-0.47866	3.31907
C	-0.73930	-1.68146	3.93712
C	-0.48384	-1.97305	5.31265
S	0.43587	-0.94376	6.34719
C	0.84750	0.40488	5.35397
C	0.43552	0.53099	3.99307
C	-1.42429	-2.67781	3.18244
C	-1.87503	-3.83187	3.76774
C	-1.65149	-4.06829	5.13731
C	-0.95371	-3.15803	5.89953
C	1.59941	1.41285	5.97822
C	1.92290	2.55487	5.28048
C	1.48418	2.73110	3.95410

C	0.75642	1.75137	3.33164
C	-6.53524	0.67381	-0.88497
C	-7.53098	-0.28741	-1.21044
C	-8.92785	-0.02547	-1.07709
S	-9.55912	1.44554	-0.43405
C	-8.16104	2.39178	-0.07981
C	-6.82707	1.96035	-0.35522
C	-7.15753	-1.59171	-1.64497
C	-8.09849	-2.52787	-1.98500
C	-9.46974	-2.22022	-1.89750
C	-9.88231	-0.98987	-1.43726
C	-8.41187	3.65601	0.47519
C	-7.36270	4.50855	0.73727
C	-6.04233	4.13260	0.42659
C	-5.78302	2.89992	-0.11287
H	0.29089	-2.75643	-4.06143
H	-0.68104	-2.59054	-6.34550
H	-2.87100	-1.49295	-6.68097
H	-0.83632	-1.71850	-2.14716
H	-4.85040	-0.41627	-5.76885
H	-6.18043	0.34994	-3.82577
H	-5.23331	0.36015	1.73007
H	-3.19413	0.00003	3.10362
H	2.26410	-0.50139	1.82862
H	3.57747	-0.05581	-0.22606
H	3.70892	0.75115	-2.51177
H	2.61740	1.70294	-4.51628
H	0.15238	2.04119	-4.54766
H	-1.23146	1.31158	-2.66135
H	-1.58087	-2.51522	2.12298
H	-2.39438	-4.57387	3.17004
H	-2.01168	-4.98412	5.59585
H	-0.75668	-3.35434	6.94960
H	1.91200	1.29064	7.01136
H	2.50324	3.33229	5.76785
H	1.71492	3.65099	3.42678
H	0.40544	1.90557	2.31795
H	-6.10518	-1.84580	-1.69503
H	-7.78622	-3.51406	-2.31276

H	-10.21124	-2.96374	-2.17318
H	-10.94035	-0.76421	-1.33894
H	-9.43357	3.95813	0.68667
H	-7.56276	5.48603	1.16534
H	-5.22783	4.82811	0.60097
H	-4.76647	2.62986	-0.37129

References

(1) Neese, F.; Wennmohs, F.; Becker, U.; Riplinger, C. The ORCA Quantum Chemistry Program Package. *J. Chem. Phys.* **2020**, *152* (22), 224108. https://doi.org/10.1063/5.0004608.

(2) Frisch, M. J.; Trucks, G. W.; Schlegel, H. B.; Scuseria, G. E.; Robb, M. A.; Cheeseman, J. R.; Scalmani, G.; Barone, V.; Petersson, G. A.; Nakatsuji, H.; Li, X.; Caricato, M.; Marenich, A. V.; Bloino, J.; Janesko, B. G.; Gomperts, R.; Mennucci, B.; Hratchian, H. P.; Ortiz, J. V.; Izmaylov, A. F.; Sonnenberg, J. L.; Williams; Ding, F.; Lipparini, F.; Egidi, F.; Goings, J.; Peng, B.; Petrone, A.; Henderson, T.; Ranasinghe, D.; Zakrzewski, V. G.; Gao, J.; Rega, N.; Zheng, G.; Liang, W.; Hada, M.; Ehara, M.; Toyota, K.; Fukuda, R.; Hasegawa, J.; Ishida, M.; Nakajima, T.; Honda, Y.; Kitao, O.; Nakai, H.; Vreven, T.; Throssell, K.; Montgomery Jr., J. A.; Peralta, J. E.; Ogliaro, F.; Bearpark, M. J.; Heyd, J. J.; Brothers, E. N.; Kudin, K. N.; Staroverov, V. N.; Keith, T. A.; Kobayashi, R.; Normand, J.; Raghavachari, K.; Rendell, A. P.; Burant, J. C.; Iyengar, S. S.; Tomasi, J.; Cossi, M.; Millam, J. M.; Klene, M.; Adamo, C.; Cammi, R.; Ochterski, J. W.; Martin, R. L.; Morokuma, K.; Farkas, O.; Foresman, J. B.; Fox, D. J. Gaussian 16 Rev. C.01, 2016.

(3) Kaiser, R. P.; Nečas, D.; Cadart, T.; Gyepes, R.; Císařová, I.; Mosinger, J.; Pospíšil, L.; Kotora, M. Straightforward Synthesis and Properties of Highly Fluorescent [5]- and [7]-Helical Dispiroindeno[2,1-c]Fluorenes. *Angew. Chem. Int. Ed.* **2019**, *58* (48), 17169–17174. https://doi.org/10.1002/anie.201908348.

(4) Cadart, T.; Nečas, D.; Kaiser, R. P.; Favereau, L.; Císařová, I.; Gyepes, R.; Hodačová, J.; Kalíková, K.; Bednárová, L.; Crassous, J.; Kotora, M. Rhodium-Catalyzed Enantioselective Synthesis of Highly Fluorescent and CPL-Active Dispiroindeno[2,1-c]Fluorenes. *Chem. – Eur. J.* **2021**, *27* (44), 11279–11284. https://doi.org/10.1002/chem.202100759.

(5) Coleman, A. C.; Areephong, J.; Vicario, J.; Meetsma, A.; Browne, W. R.; Feringa, B. L. In Situ Generation of Wavelength-Shifting Donor–Acceptor Mixed-Monolayer-Modified Surfaces. *Angew. Chem. Int. Ed.* **2010**, *49* (37), 6580–6584. https://doi.org/10.1002/anie.201002939.

(6) Bruker, APEX4, SAINT and SADABS. Bruker AXS Inc., Madison, Wisconsin, USA.

(7) Sheldrick, G. M. SHELXT – Integrated Space-Group and Crystal-Structure Determination. *Acta Crystallogr. Sect. Found. Adv.* **2015**, *71* (1), 3–8. https://doi.org/10.1107/S2053273314026370.

(8) Sheldrick, G. M. A Short History of SHELX. *Acta Crystallogr. A* **2008**, *64* (1), 112–122. https://doi.org/10.1107/S0108767307043930.

(9) Dolomanov, O. V.; Bourhis, L. J.; Gildea, R. J.; Howard, J. a. K.; Puschmann, H. OLEX2: A Complete Structure Solution, Refinement and Analysis Program. *J. Appl. Crystallogr.* **2009**, *42* (2), 339–341. https://doi.org/10.1107/S0021889808042726.

(10) Spek, A. L. PLATON SQUEEZE: A Tool for the Calculation of the Disordered Solvent Contribution to the Calculated Structure Factors. *Acta Crystallogr. Sect. C Struct. Chem.* **2015**, *71* (1), 9–18. https://doi.org/10.1107/S2053229614024929.

(11) Fukuda, K.; Nagami, T.; Fujiyoshi, J.; Nakano, M. Interplay between Open-Shell Character, Aromaticity, and Second Hyperpolarizabilities in Indenofluorenes. *J.*

Phys. Chem. A **2015**, *119* (42), 10620–10627. https://doi.org/10.1021/acs.jpca.5b08520.
(12) Grimme, S.; Hansen, A.; Ehlert, S.; Mewes, J.-M. r2SCAN-3c: A “Swiss Army Knife” Composite Electronic-Structure Method. *J. Chem. Phys.* **2021**, *154* (6), 064103. https://doi.org/10.1063/5.0040021.
(13) Barone, V.; Cossi, M. Quantum Calculation of Molecular Energies and Energy Gradients in Solution by a Conductor Solvent Model. *J. Phys. Chem. A* **1998**, *102* (11), 1995–2001. https://doi.org/10.1021/jp9716997.
(14) Rahalkar, A.; Stanger, A. “Aroma”, Http://Chemistry.Technion.Ac.Il/Members/Amnon-Stanger/ (accessed 2024-03-31).
(15) Stanger, A. Nucleus-Independent Chemical Shifts (NICS): Distance Dependence and Revised Criteria for Aromaticity and Antiaromaticity. *J. Org. Chem.* **2006**, *71* (3), 883–893. https://doi.org/10.1021/jo051746o.
(16) Stanger, A. Obtaining Relative Induced Ring Currents Quantitatively from NICS. *J. Org. Chem.* **2010**, *75* (7), 2281–2288. https://doi.org/10.1021/jo1000753.
(17) Gershoni-Poranne, R.; Stanger, A. The NICS-XY-Scan: Identification of Local and Global Ring Currents in Multi-Ring Systems. *Chem. – Eur. J.* **2014**, *20* (19), 5673–5688. https://doi.org/10.1002/chem.201304307.
(18) Becke, A. D. Density-functional Thermochemistry. III. The Role of Exact Exchange. *J. Chem. Phys.* **1993**, *98* (7), 5648–5652. https://doi.org/10.1063/1.464913.
(19) Hehre, W. J.; Ditchfield, R.; Pople, J. A. Self—Consistent Molecular Orbital Methods. XII. Further Extensions of Gaussian—Type Basis Sets for Use in Molecular Orbital Studies of Organic Molecules. *J. Chem. Phys.* **1972**, *56* (5), 2257–2261. https://doi.org/10.1063/1.1677527.
(20) Krishnan, R.; Binkley, J. S.; Seeger, R.; Pople, J. A. Self-consistent Molecular Orbital Methods. XX. A Basis Set for Correlated Wave Functions. *J. Chem. Phys.* **1980**, *72* (1), 650–654. https://doi.org/10.1063/1.438955.
(21) Ishigaki, Y.; Mizuno, S.; Sugawara, K.; Hashimoto, T.; Suzuki, S.; Suzuki, T. Thermal Equilibrium between Quinoid/Biradical Forms Enhancing Electrochemical Amphotericity. *Chem. – Eur. J.* **2024**, e202400916. https://doi.org/10.1002/chem.202400916.
(22) Harimoto, T.; Tadokoro, T.; Sugiyama, S.; Suzuki, T.; Ishigaki, Y. Domino-Redox Reaction Induced by An Electrochemically Triggered Conformational Change. *Angew. Chem.* **2024**, *136* (1), e202316753. https://doi.org/10.1002/ange.202316753.
